# Dao-Chi Powder Ameliorates Pancreatitis-Induced Intestinal and Cardiac Injuries *via* Regulating the Nrf2-HO-1-HMGB1 Signaling Pathway in Rats

**DOI:** 10.3389/fphar.2022.922130

**Published:** 2022-07-11

**Authors:** Jiaqi Yao, Yifan Miao, Yumei Zhang, Lv Zhu, Huan Chen, Xiajia Wu, Yue Yang, Xiaoyu Dai, Qian Hu, Meihua Wan, Wenfu Tang

**Affiliations:** ^1^ Department of Integrated Traditional Chinese and Western Medicine, West China Hospital, Sichuan University, Chengdu, China; ^2^ Department of Traditional Chinese Medicine, Xiang’an Hospital of Xiamen University, School of Medicine, Xiamen University, Xiamen, China; ^3^ Clinical Trial Center, National Medical Products Administration Key Laboratory for Clinical Research and Evaluation of Innovative Drugs, West China Hospital, Sichuan University, Chengdu, China

**Keywords:** severe acute pancreatitis, Dao-Chi powder, oxidative stress, inflammatory responses, Nrf2/HO-1/HMGB1

## Abstract

Dao-Chi powder (DCP) has been widely used in the treatment of inflammatory diseases in the clinical practice of traditional Chinese medicine, but has not been used in acute pancreatitis (AP). This study aimed to evaluate the effect of DCP on severe AP (SAP) and SAP-associated intestinal and cardiac injuries. To this end, an SAP animal model was established by retrograde injection of 3.5% taurocholic acid sodium salt into the biliopancreatic ducts of rats. Intragastric DCP (9.6 g/kg.BW) was administered 12 h after modeling. The pancreas, duodenum, colon, heart and blood samples were collected 36 h after the operation for histological and biochemical detection. The tissue distributions of the DCP components were determined and compared between the sham and the SAP groups. Moreover, molecular docking analysis was employed to investigate the interactions between the potential active components of DCP and its targets (Nrf2, HO-1, and HMGB1). Consequently, DCP treatment decreased the serum levels of amylase and the markers of gastrointestinal and cardiac injury, further alleviating the pathological damage in the pancreas, duodenum, colon, and heart of rats with SAP. Mechanistically, DCP rebalanced the pro-/anti-inflammatory cytokines and inhibited MPO activity and MDA levels in these tissues. Furthermore, Western blot and RT-PCR results showed that DCP intervention enhanced the expression of Nrf2 and HO-1 in the duodenum and colon of rats with SAP, while inhibiting the expression of HMGB1 in the duodenum and heart. HPLC-MS/MS analysis revealed that SAP promoted the distribution of ajugol and oleanolic acid to the duodenum, whereas it inhibited the distribution of liquiritigenin to the heart and ajugol to the colon. Molecular docking analysis confirmed that the six screened components of DCP had relatively good binding affinity with Nrf2, HO-1, and HMGB1. Among these, oleanolic acid had the highest affinity for HO-1. Altogether, DCP could alleviated SAP-induced intestinal and cardiac injuries *via* inhibiting the inflammatory responses and oxidative stress partially through regulating the Nrf2/HO-1/HMGB1 signaling pathway, thereby providing additional supportive evidence for the clinical treatment of SAP.

## Introduction

Acute pancreatitis (AP) is an acute inflammatory disease of the pancreas and peripancreatic tissues. The incidence of AP is estimated at 110 to 140 per 100 000 population, with an estimated overall mortality of approximately 1% (Xiao et al., 2016; Sellers et al., 2018; Garg et al., 2019); however, in patients with severe AP (SAP) with pancreatic necrosis or organ failure (lung, kidney, heart, etc.), mortality may be as high as 30%–40% (Petrov et al., 2010; Boxhoorn et al., 2020). After the onset of AP, the excessive release of inflammatory cytokines primarily causes acute gastrointestinal injury and the subsequent disruption of the integrity of the intestinal mucosa, leading to the translocation of bacteria and endotoxins (ET), ultimately leading to the amplification of the inflammatory response and secondary infection (Dellinger et al., 2012; Banks et al., 2013). Without timely treatment, the inflammatory reaction spreads to the heart and ultimately affects cardiac function, greatly increasing the early mortality associated with SAP (Klingensmith and Coopersmith, 2016). Therefore, effective interventions targeting intestinal and cardiac injuries might reduce the mortality associated with SAP, and the exploration of new treatment measures is crucial for the clinical treatment of SAP.

Oxidative stress is an unstable state caused by the disruption of the balance between the oxidative and antioxidant systems in the body. Oxidative stress and redox status are involved in the onset of AP and contribute to the local and systemic inflammatory responses during AP (Pérez et al., 2015). Nuclear factor erythroid 2-related factor 2 (Nrf2) is an important transcription factor that is widely expressed in the intestines, heart, brain, and pancreas, and can regulate the expression of a large number of detoxification and antioxidant genes (Kensler et al., 2007). Heme oxygenase-1 (HO-1) is an important inducible antioxidant enzyme regulated by Nrf2 (Zhang et al., 2011; Dodson et al., 2019). Studies have shown that Nrf2 regulates the physiological function of mitochondria, DNA damage repair, and inflammatory reactions (Tebay et al., 2015; Ahmed et al., 2017; Mohan and Gupta, 2018), which are involved in the development of many inflammatory diseases, including AP (Keleku-Lukwete et al., 2018). A variety of drugs, including melatonin, can protect against pancreatic damage by activating Nrf2/HO-1 signaling (Jung et al., 2010; Wang Y. Z. et al., 2014b). The activation of Nrf2 and HO-1 has been demonstrated to protect against inflammatory intestinal injury, such as intestinal ischemia/reperfusion (I/R) injury (Zu et al., 2018; Jin et al., 2019) and septic intestinal injury (Yu et al., 2017). Additionally, the up-regulation of Nrf2 through drug intervention (e.g., berbamine) can also inhibit oxidative stress and inflammation to alleviate cardiac injury (Xingyue et al., 2021; Hao et al., 2022; Xu et al., 2022).

High Mobility Group Box-1 (HMGB1) is a specific non-histone nucleoprotein in eukaryotic cells, involved in mediating various inflammatory diseases (Kang et al., 2014). Moreover, oxidative stress itself can induce the active and passive release of pro-inflammatory alarmin HMGB1, and Nrf2/HO-1 induction plays a protective role in several disease states by suppressing the secretion of HMGB1 and decreasing HMGB1 expression (Abraham and Kappas, 2005; Tang et al., 2007). The role of the Nrf2/HO-1/HMGB1 pathway has received considerable attention in studies of the mechanisms of many diseases (Wang J. et al., 2014a; Wang et al., 2015; Yu et al., 2017). Elevated circulating HMGB1 levels in patients with AP have been reported to serve as a bridge between intestinal bacterial migration and systemic inflammation, inducing multiple organ damage (Yang et al., 2017). Hydrogen gas has been shown to improve septic intestinal injury by regulating the Nrf2/HO-1/HMGB1 pathway (Yu et al., 2017). Another study also pointed out that the β-adrenergic receptor ameliorated myocardial I/R injury by regulating the Nrf2/HO-1/HMGB1 axis in rats (Wang J. et al., 2014a). Consequently, a promising strategy for alleviating SAP-associated intestinal and cardiac injuries is to regulate oxidative stress and inflammatory responses *via* the Nrf2/HO-1/HMGB1 pathway.

Dao-Chi powder (DCP) comes from a classic TCM book called Key to Therapeutics of Children’s Disease. It is composed of four traditional Chinese herbs: *Rehmannia glutinosa* (Gaertn.) DC. [Orobanchaceae; radix rehmanniae] (6g), *Clematis armandi* Franch. [Ranunculaceae; caulis clematidis armandii] (6g), *Lophatherum gracile* Brongn. [Poaceae; lophatheri herba], and *Glycyrrhiza uralensis* Fisch. ex DC. [Fabaceae; glycyrrhizae radix et rhizoma] (6g), and indications for relieving oral ulcer in children (Luo et al., 2016). Accumulating evidence has revealed that DCP displays potent anti-inflammatory and antioxidant activities. For instance, Yang et al. (2019) reported that DCP combined with ribavirin effectively reduced serum inflammatory factor levels in children with hand-foot-mouth disease (Yang et al., 2019). Yin and Nie (2017) showed that supplementation with DCP combined with vitamin B_12_ inhibited oxidative stress and reduced serum pro-inflammatory mediators in rats with recurrent oral ulcers (Yin and Nie, 2017). These studies have shed light on the application of DCP in inflammatory diseases.

However, although DCP has been widely used in clinical practice, almost all studies on DCP have been reported in China, and few studies have uncovered the therapeutic effect of DCP on SAP. This study is the first to evaluate the potential protective effect of DCP on intestinal and cardiac injuries related to SAP and further elucidate the underlying mechanism and material basis, providing a basis for future studies and clinical application of DCP against SAP.

## Materials and Methods

### Reagents and Antibodies

Taurocholic acid sodium salt (NaT, #909688) was purchased from J&K Scientific, Ltd. (Beijing, China). ELISA kits, including interleukin-6 (IL-6, #ERC003.96) and interleukin-10 (IL-10, #ERC004.96) kits, were purchased from Neobioscience Technology Company (Shenzhen, China). D-Lactate (D-Lac, #H263-1-2), vasoactive intestinal peptide (VIP, #H219), and motilin (MTL, #H182), and other assay kits, including ET (#E039-1-1), malondialdehyde (MDA, #A003), and myeloperoxidase (MPO, #A044) were obtained from Nanjing Jiancheng Bioengineering Institute (Nanjing, China). The anti-Nrf2 (#ab89443), anti-HO-1 (#ab68477), and anti-HMGB1 (#ab79823) were purchased from Abcam (Cambridge, Massachusetts, United Kingdom). Anti-GAPDH (#10494-1-AP) and anti-β-actin (#AC026) antibodies were purchased from Proteintech (Rosemont, IL, United States) and ABclonal (Wuhan, China), respectively. Goat anti-mouse and anti-rabbit IgG-HRP (#31430 and #31460, respectively) were purchased from Thermo Fisher Scientific (Waltham, MA, United States). All reference standards were purchased from Beijing Zhongkezhijian Biotechnology Co., Ltd. (Beijing, China); detailed information is provided in Table 1. The methanol and ammonium acetate used for high-performance liquid chromatography (HPLC) were sourced from Thermo Fisher Scientific (A452-4 and 033440, respectively). All other reagents were obtained from a standard source and were of analytical grade.

**TABLE 1:** Reference standards information.

Standard name	Purity (HPLC)	Product No.	Chemical structure
Ajugol	≥98%	52949-83-4	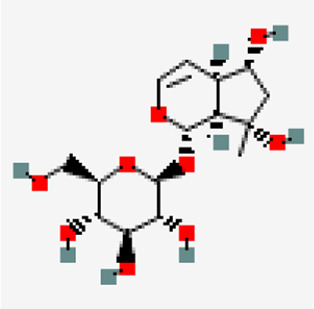
Catalpol	≥98%	2415-24-9	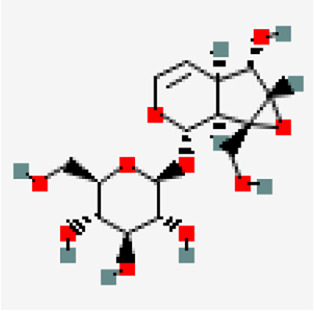
Martynoside D	≥98%	81720-08-3	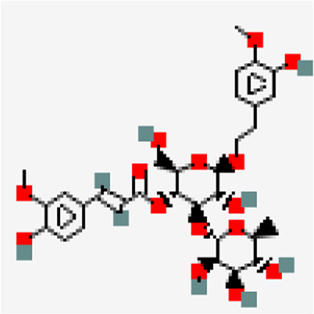
Oleanolic acid	≥98%	508-02-1	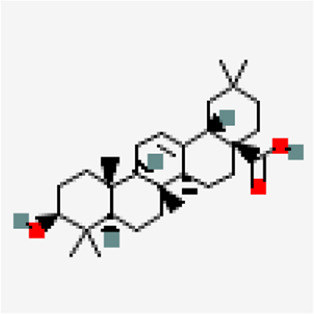
Stigmasterol	≥98%	83-48-7	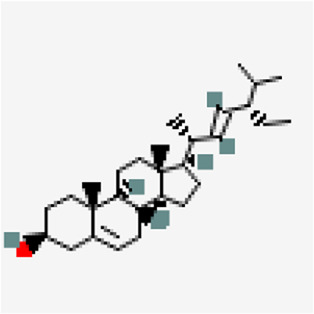
Beta-Sitosterol	≥98%	83-46-5	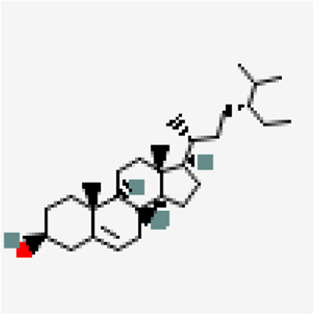
Isoorientin	≥98%	4261-42-1	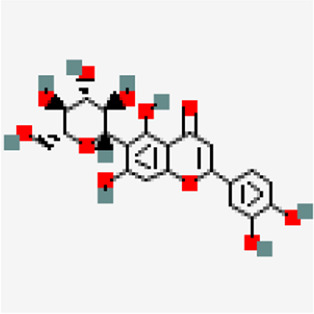
Chlorogenic acid	≥98%	327-97-9	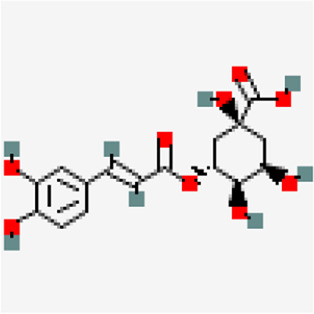
Glycyrrhizic acid	≥98%	1405-86-3	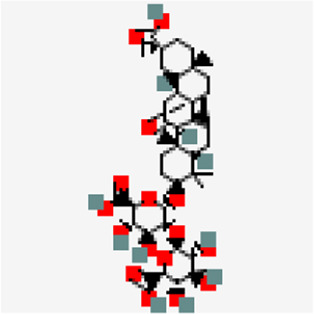
Liquiritigenin	≥98%	578-86-9	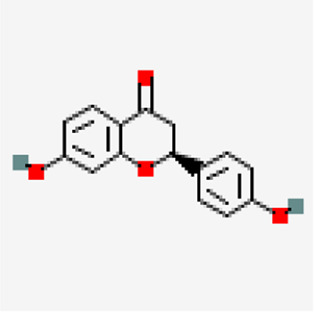

### Dao-Chi Powder Preparation

The lyophilized powders of these herbs (40:1, i.e., 1 g extract/40 g herb) used in the current experiment were purchased from Chengdu Green Herbal Pharmaceutical Co. Ltd. (Chengdu, China), and the detailed composition is shown in Table 2. The procedure for synthesizing the DCP extract was as follows: 24g herbal constituents of DCP was soaked in 600 ml sterile filtered water for 30 min and then was boiled for 30 min to a concentrated solution (∼120 ml). The concentrated liquid was then removed from the dregs and stored separately. Another 600 ml water was added to the dregs then boiled and concentrated again as described above. The twice extracted solution was combined (∼240 ml) and lyophilized into powder (∼0.6g) using an EYELA FDU-2110 lyophilizer (Tokyo Rikakikai Co., Ltd.; Tokyo, Japan) and stored at −20°C. The suggested dosage of DCP is 24g per child (10–30 Kg.BW) per day, indicating a dose range of 0.8–2.4 g/kg.BW for humans (Luo et al., 2016; Yang et al., 2019). Meanwhile, the equivalent dose for rats was calculated based on the recommended dosage for humans with the conversion coefficient of 6 and the daily dose was at a range of 4.8–14.4 g/kg.BW (Nair and Jacob, 2016; Zhang et al., 2017). On this basis, our further pharmacokinetic study confirmed that the main components of DCP could be accurately quantified by HPLC-MS/MS at the dose of 9.6 g/kg.BW, while the other two relatively low doses could not be detected (data presented in the Supplementary Table S1). Finally, the lyophilised powder of DCP was dissolved in sterile double-distilled water to a concentration of 0.96 g crude drug per mL, and administered orally to the rats at a dose of 9.6 g/kg.BW for once.

**TABLE 2:** The ingredients list of Dao-Chi powder.

Ingredients	Chinese name	Plant parts	Weight (g)	Batch No.
*Rehmannia glutinosa* (Gaertn.) DC.	Shengdihuang	Root	6	18020036
*Clematis armandi* Franch	Chuanmutong	Stem	6	17110035
*Lophatherum gracile* Brongn	Danzhuye	Stem and leaf	6	17090016
*Glycyrrhiza uralensis* Fisch. ex DC.	Shenggancao	Root and rhizome	6	18010077

### Ethics and Animals

All animal procedures were approved by the Animal Ethics Committee of the West China Hospital, Sichuan University (No. 2018139A). Male Sprague-Dawley rats (*n* = 40, 300 ± 20 g) were purchased from Chengdu Dashuo Biological Technology Co., Ltd. (Chengdu, China; production license No. SCXK (Sichuan) 2017-24). The rats were fed adaptively for 1 week under standard feeding conditions and fasted for 12 h before the experiment.

### Induction of Severe AP and Intervention

Part 1: The rats were randomly divided into three groups (*n* = 8): sham operation (sham), SAP, and DCP. As previously described (Zhu et al., 2017), 3.5% NaT (1 ml/kg.BW) was used to induce SAP by retrograde infusion into the biliopancreatic duct at a rate of 6 ml/h. Rats in the sham group underwent the same procedure but were injected with sterile saline. Next, the intragastric DCP (9.6 g/kg. BW) was administered to rats 12 h after modeling, and rats in the sham and SAP groups were administered the same dose of sterile saline. Finally, the rats were euthanized with a 2% sodium pentobarbital overdose (200 mg/kg).BW, intraperitoneal injection). The pancreas, duodenum, colon, and heart, were harvested and blood samples were collected 36 h after the operation for histological and biochemical detection, as described below.

Part 2: The rats were randomly divided into two groups (*n* = 6): sham + DCP group and SAP + DCP group. The experimental SAP and sham operations were the same as those described in Part 1. Rats in the two groups were orally administered DCP (9.6 g/kg. BW) 12 h after the operation and sacrificed 24 h after administration. The duodenum, colon, and cardiac tissues were harvested at 24 h after the operation for HPLC-MS/MS analysis, as described below.

### Histopathology

Neutral 10% formalin-fixed organ tissue samples were embedded (paraffin, sliced (5 µM), and further stained with hematoxylin and eosin. Slides for the pancreas, duodenum, colon, and heart were observed under an inverted microscope (Axio Observer.Z1, Zeiss, Germany) and scored by a professional pathologist in a blinded manner. Pancreatic histopathology was determined according to Kusske et al. (1996) (Kusske et al., 1996). (points 0–4, edema, inflammation, hemorrhage, and necrosis). Duodenal mucosal lesions were graded using the scoring system described by Chiu et al. (1970) (Chiu et al., 1970). The colon was examined for edema, inflammation, necrosis, and hyperemia/hemorrhage (points 0–4). For the heart, histopathological scoring was evaluated based on a 0–4 scoring method as described previously (Laster et al., 1994), and the parameters included interstitial edema, hemorrhage, and neutrophil infiltration. The total histopathogical score is the mean of the combined scores for each parameter from both investigators.

### Measurement of Serum Amylase, Cardiac Troponin I, and Creatine Kinase-MB Levels

The supernatants of blood samples were collected after centrifugation (3,000 rpm, 10 min). An automatic biochemical analyzer (7170A, HITACHI, Tokyo, Japan) was used to determine the levels of amylase, cTnI, and CK-MB within the supernatants.

### Measurement of Serum Endotoxins Levels

The supernatants of blood samples were collected after centrifugation (3,000 rpm, 10 min). Next, the ET levels in the supernatants were estimated using a Limulus Amoebocyte Lysate Assay Kit according to the manufacturer’s instructions.

### ELISA Analysis

The supernatants of blood samples were collected after centrifugation (3,000 rpm, 10 min), and the levels of D-Lac, VIP, MTL, and inflammatory mediators (IL6 and IL-10) within the supernatants were detected using the corresponding ELISA kits, according to the manufacturer’s instructions.

### Tissue MDA and MPO Analysis

The supernatants of the duodenum, colon, and cardiac tissue samples were obtained by cold homogenization and centrifugation (13000 rpm, 10 min), and the levels of MDA and MPO in the supernatants were detected using the corresponding testing kit according to the manufacturer’s instructions.

### Western Blot Analysis

Total proteins from duodenum, colon, and heart were extracted using lysis buffer containing 1% Triton X-100, 1% SDS, and 1% protease inhibitors and quantified using BCA Kit (#P0012S; Beyotime, Shanghai, China). Equivalent amounts of protein were separated by sodium dodecyl sulfate-polyacrylamide gel electrophoresis and transferred onto nitrocellulose membrane filters. The membranes were blocked with protein-free rapid blocking buffer (×5) (#PS108; Epizyme Biomedical Technology Co., Ltd.; Shanghai, China) in TBST for 1 h at room temperature and then probed with antibodies against Nrf2 (1:1000), HO-1 (1:1000), HMGB1 (1:1000), GAPDH (1:5000), and β-actin (1:5000) at 4°C overnight. Membranes were washed with Tween20/TBS before incubated with the appropriate secondary antibody. After washing, the membranes were visualized using a chemiluminescence detection kit (#P0018S; Beyotime, Shanghai, China). Corresponding protein grayscale statistics were quantified using NIH ImageJ 1.47 software.

### Real-Time PCR Analysis

Total RNA from the duodenum, colon, and heart was extracted with TRIzol reagent (#15596026, Thermo Fisher Scientific, Waltham, MA, United States) and quantified using NanoDrop 2000. RNA was reverse-transcribed into cDNA using a reverse transcription kit (#RR047A, TaKaRa, Shiga, Japan). RT-PCR was performed using TB Green™ Premix Ex Taq™ II (#RR820A, TaKaRa, Shiga, Japan), and detection was performed using a CFX96 Touch System (Bio-Rad, Hercules, CA, United States). GAPDH was used as a housekeeping gene. The primers used in this study are listed in Table 3.

**TABLE 3:** RT-PCR primer sequences.

Genes	Primer pairs	Product length (bp)
NFE2L2	F: 5′-TCT​TGG​GGT​AAG​TCG​AGA​AGT​GT-3′	140
	R: 5′-GTT​GTA​ACT​GAG​CGA​AAA​AGG​C-3′	
HMOX1	F: 5′-GTG​CAG​AGA​ATT​CTG​AGT​TCA-3′	100
	R: 5′-GCC​GTA​TAG​ATA​TGG​TAC​AAG​GA-3′	
HMGB1	F: 5′-TAT​GGC​AAA​GGC​TGA​CAA​GG-3′	196
	R: 5′-CTT​CGC​AAC​ATC​ACC​AAT​GGA-3′	
GAPDH	F: 5′-GGT​GAA​GGT​CGG​TGT​GAA​CG-3′	233
	R: 5′-CTC​GCT​CCT​GGA​AGA​TGG​TG-3′	

### High-Performance Liquid Chromatography-MS/MS Analysis

The main components of DCP in duodenum, colon, and cardiac tissue homogenates (10%) were detected by a Shimadzu UPLC 30A System (Shimadzu, Kyoto, Japan) coupled with an AB4500 triple quadrupole instrument (AB Sciex, Ontario, Canada), while data was processed by Analyst 1.6.3 software (AB Sciex, Ontario, Canada). Chromatographic separation was performed on a SUPELCO column (3 μM, 50 mm × 2.1 μM) at 40°C. Gradient elution of methanol (A) and 5 mmol/L ammonium acetate (B) at a flow rate of 0.3 ml/min was employed: 0–0.2 min, 10% A; 0.2–5 min, 10–100% A; 5–8 min, 100% A; 8–8.01 min, 100–10% A; 8.01–10 min, 10% A. The injection volume was 5 μl. The AB4500 system was manipulated under the multiple reaction monitoring mode and ionized in negative ion mode using electrospray. The parameters were as follows: mass range, 50–1200 m/z; capillary voltage, 1.0 KV; source temperature, 120°C; desolvation temperature, 400°C; cone gas flow, 50 L/h; desolvation gas flow, 800 L/h.

### Molecular Docking

Molecular docking analysis was employed to verify the binding affinity and interactions between the DCP components and targets (Nrf2, HO-1, and HMGB1). The 2D structures of the DCP components were downloaded from the PubChem database (https://pubchem.ncbi.nlm.nih.gov/) and the 3D structures of Nrf2, HO-1, and HMGB1 were screened from the RCSB Protein Data Bank (PDB) (https://www.rcsb.org/). The docking of each compound with the target and calculation of the corresponding free binding energies was performed using AutoDock Tools (Version 1.5.6) and AutoDock Vina (version 1.1.2). The interactions and binding modes of the most tightly bound set of molecules and targets were visualized using PyMOL (version 2.3) after docking.

### Statistical Analysis

GraphPad Prism 8.3.1 software (GraphPad Prism software Inc., San Diego, CA, United States) was adopted to perform the statistical analyses. Multiple groups were compared by one-way ANOVA followed by Dunnett’s post-hoc test. All values are presented as mean ± SEM and statistical significance was set at *p <* 0.05.

## Results

### Dao-Chi Powder Attenuated the Pathological Damage of Pancreatic Tissues in Rats With Severe AP

**FIGURE 1:**
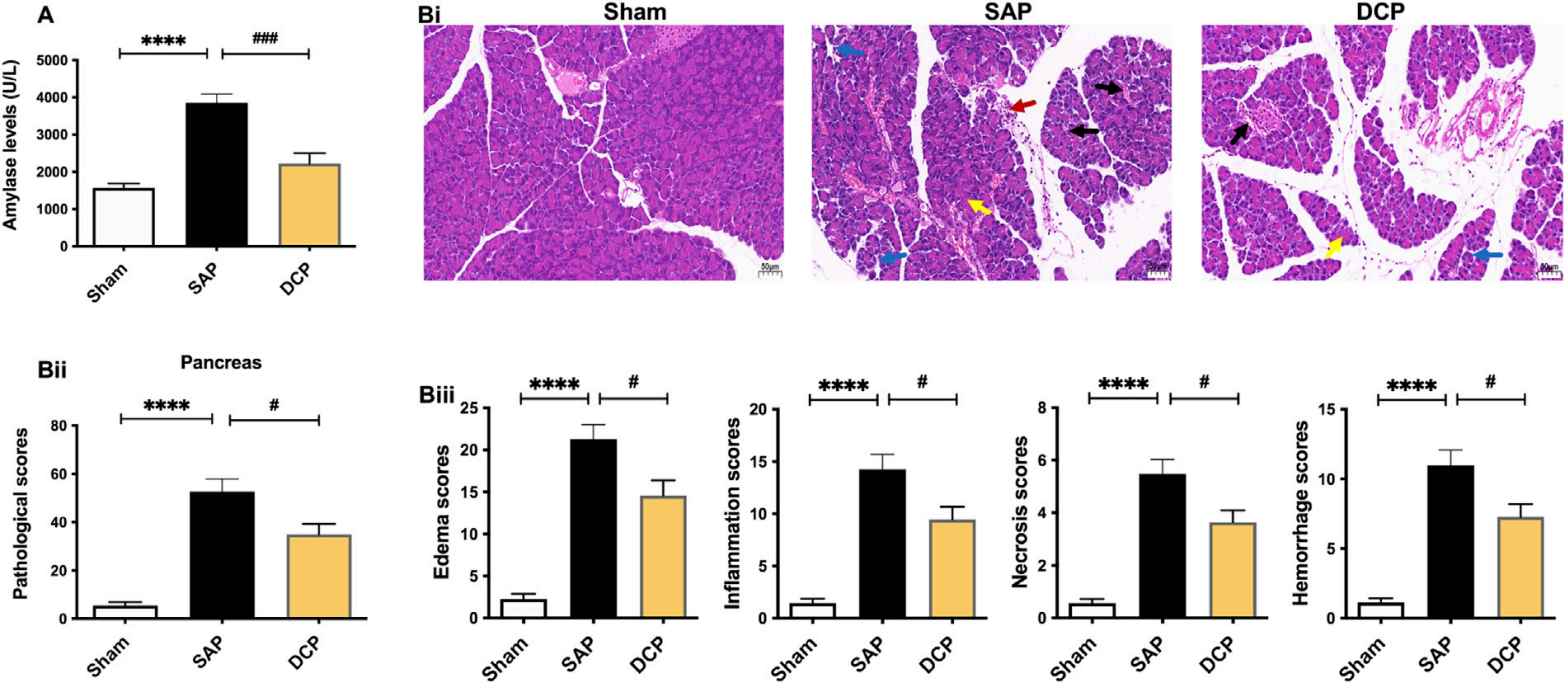
DCP attenuated the pathological damage of pancreatic tissues in rats with SAP. **(A)**: Serum amylase levels in Sham, SAP and DCP group; **(Bi)**: Representative pathological images of H & E staining on pancreatic tissues (× 200), arrows (blue, red, yellow, black) indicated pancreatic edema, inflammation, necrosis, and hemorrhage respectively; **(Bii)**: Total pathological scores of the pancreatic tissues of each group. **(Biii)**: Histological scores of edema, inflammation, necrosis and hemorrhage of pancreas in each group. The results are presented as the mean ± SEM. ****p < 0.0001 vs. sham group, ^#^ *p <* 0.05, ^###^ *p <* 0.005 vs. SAP group; ns, *p >* 0.05. sham: sham operation group; SAP: Sever acute pancreatitis group; DCP: Dao-Chi powder treatment group.

In this study, a rat SAP model was established by injecting NaT into the biliopancreatic duct. Elevated serum amylase level is a common diagnostic criterion for AP clinically (Rompianesi et al., 2017). As shown in Figure 1A, the serum amylase levels in the SAP group were much higher than those in the sham group (*p <* 0.001), whereas they were down-regulated by DCP administration (*p <* 0.005). Consistently, H & E staining results showed obvious tissue edema, neutrophil infiltration, hemorrhage, and marked necrosis in the pancreas of rats with SAP (Figure 1Bi), indicating that the SAP models were established successfully. DCP treatment distinctly attenuated the pancreatic pathological injury, with reduced pathological scores of total edema, inflammation, necrosis, hemorrhage (all *p <* 0.05; Figures 1Bi,Biii). Taken together, the above results suggest that DCP can alleviate pathological damage of pancreas in rats with NaT-induced SAP.

### Dao-Chi Powder Reduced the Markers of Gastrointestinal and Cardiac Injuries in Serum of Severe AP Rats

**FIGURE 2:**
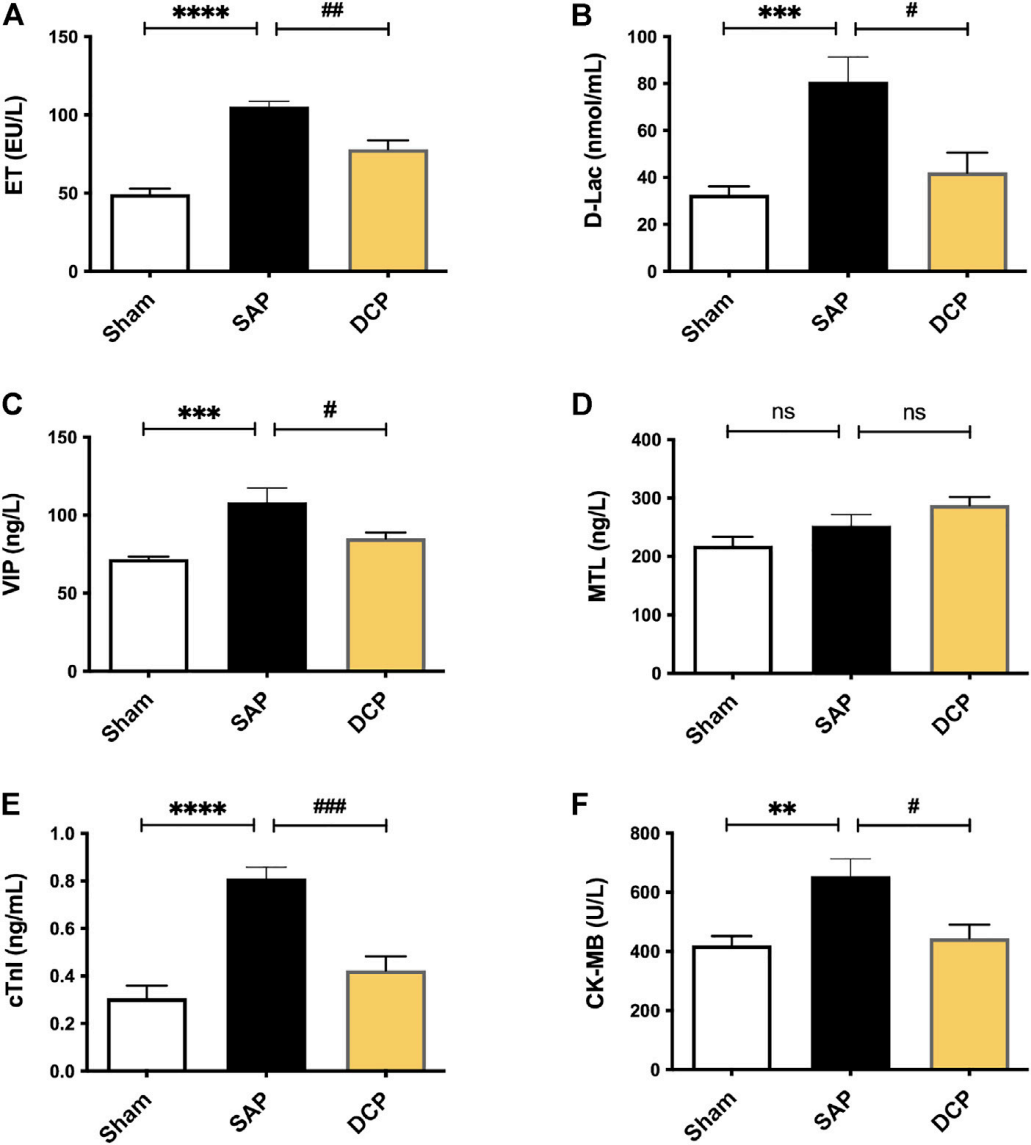
DCP reduced the markers of gastrointestinal and cardiac injuries in serum. **(A–F)**: Serum levels of ET, D-Lac, VIP, MTL, cTnI and CK-MB in rats from Sham, SAP and DCP group. The results are presented as the mean ± SEM. **p < 0.01, ***p < 0.005, ****p < 0.0001 vs. sham group, ^#^ *p <* 0.05, ^##^ *p <* 0.01, ^###^ *p <* 0.005 vs. SAP group; ns, *p >* 0.05. sham: sham operation group; SAP: Sever acute pancreatitis group; DCP: Dao-Chi powder treatment group.

Moreover, serum markers of gastrointestinal impairment such as ET, D-Lac, VIP, and MTL were also determined. According to the results, the levels of ET, D-Lac, and VIP in serum were significantly elevated in the SAP group (*p <* 0.001, *p <* 0.005, *p <* 0.005, respectively; Figures 2A–C), which were decreased after DCP administration (*p <* 0.01, *p <* 0.05, *p <* 0.05, respectively; Figures 2A–C), and DCP administration slightly up-regulated the serum MTL levels compared with the SAP group (Figure 2D). Moreover, compared with the SAP group, related cardiac function parameters, such as cTnI and CK-MB, were significantly lowered following DCP intervention (*p <* 0.005, *p <* 0.05; Figures 2E,F).

### Dao-Chi Powder Ameliorated the Pathological Damage of Intestinal and Cardiac Tissues in Rats With Severe AP

**FIGURE 3:**
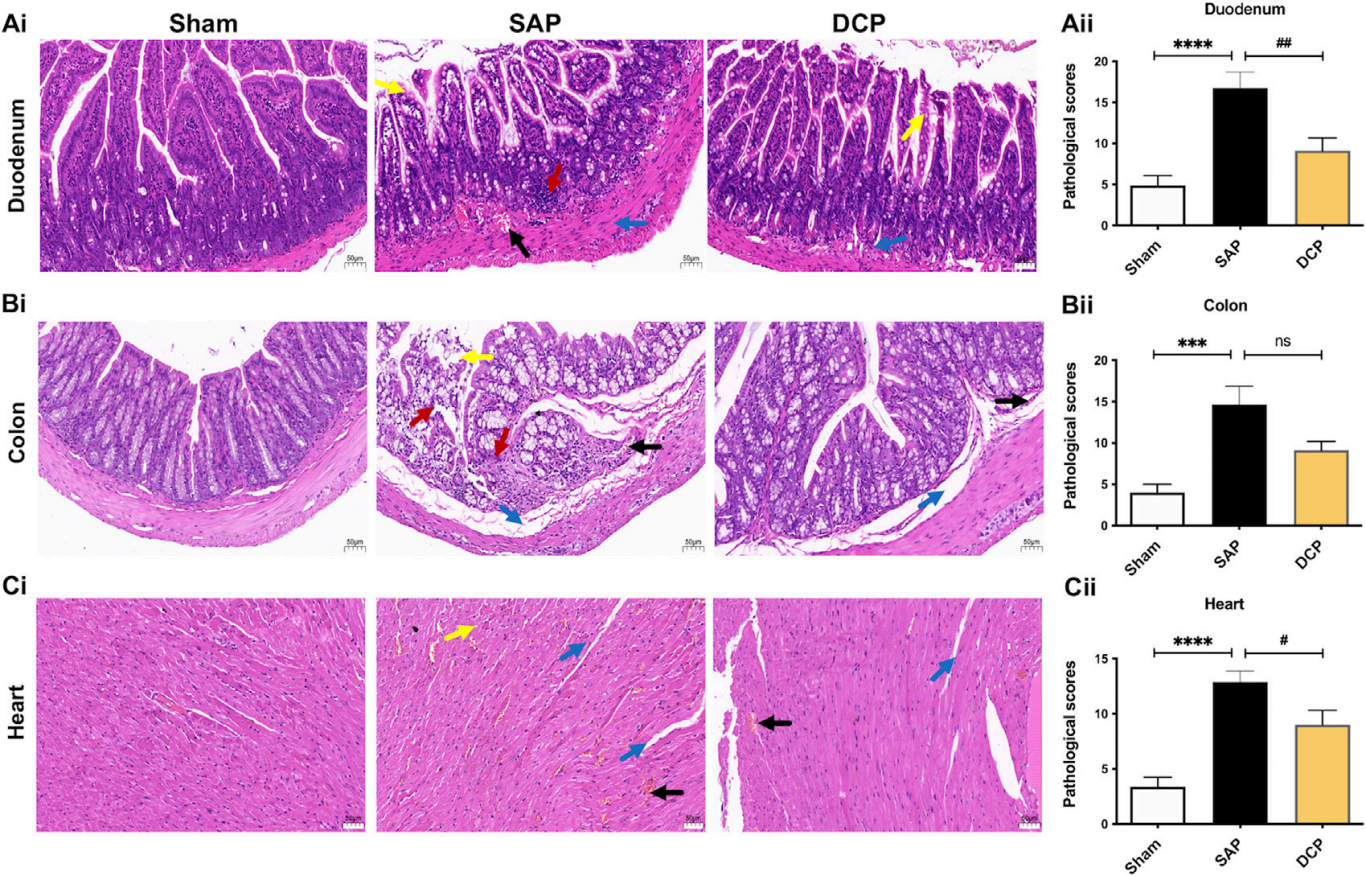
DCP ameliorated the pathological damage of intestinal and cardiac tissues in rats with SAP. **(Ai,Aii)**: Representative images of H & E staining (× 200) on duodenum and corresponding pathological scores of rats in sham, SAP and DCP group; **(Bi,Bii)**: Representative images of H & E staining (× 200) on colon and corresponding pathological scores of each group; **(Ci,Cii)**: Representative images of H & E staining (× 200) on heart and corresponding pathological scores of each group. Arrows (blue, red, yellow, black) indicated edema, inflammation, necrosis, and hemorrhage respectively. The results are presented as the mean ± SEM. ***p < 0.005, ****p < 0.0001 vs. sham group, ^**#**^ *p <* 0.05, ^**##**^ *p <* 0.01 vs. SAP group; ns, *p >* 0.05. sham: sham operation group; SAP: Sever acute pancreatitis group; DCP: Dao-Chi powder treatment group.

To investigate the therapeutic role of DCP in intestinal and cardiac injuries in rats with SAP, H & E staining of the duodenum, colon, and cardiac tissues was performed. The results revealed higher pathological scores in the duodenum in rats with SAP, with more pronounced edema, neutrophil infiltration, telangiectasia, and hyperemia (*p <* 0.001; Figures 3Ai,Aii). Notably, DCP treatment effectively mitigated duodenal injury and lowered pathological scores (*p <* 0.01; Figures 3Ai,Aii). Meanwhile, obvious pathological changes were also observed in the colon of rats with SAP, characterized by infiltration of inflammatory cells and hemorrhage in the glands, widening of the submucosal space, and necrosis of intestinal villi, accompanied by increased pathological scores (*p <* 0.005; Figures 3Bi,Bii). Similarly, DCP administration ameliorated colon injury and slightly altered pathological scores (Figures 3Bi,Bii). Consistently, H & E staining showed marked inflammatory cell infiltration, tissue edema, and necrosis in cardiac tissues of rats with SAP, together with increased pathological scores (*p <* 0.001; Figures 3Ci,Cii). DCP protected against SAP-related cardiac injury and decreased pathological scores (*p <* 0.05; Figures 3Ci,Cii). These results provide convincing evidence that DCP ameliorates the pathological damage to intestinal and cardiac tissues in rats with NaT-induced SAP.

### Dao-Chi Powder Inhibited Inflammatory Responses and Oxidative Stress in Intestinal and Cardiac Tissues of Rats With Severe AP

**FIGURE 4:**
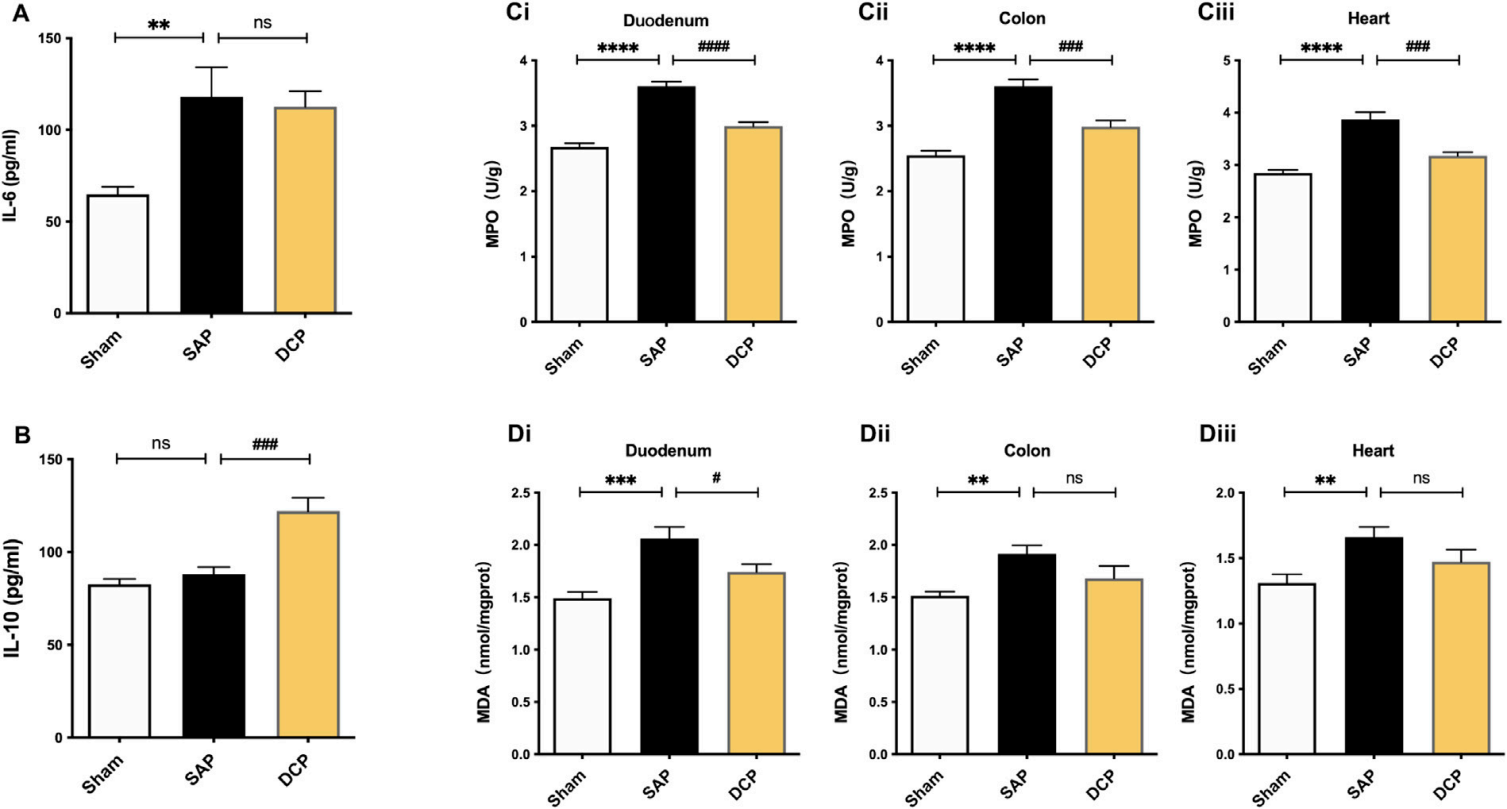
DCP inhibited inflammatory responses and oxidative stress in intestinal and cardiac tissues of rats with SAP. **(A)** Serum IL-6 levels of rats in sham, SAP and DCP group; **(B)** Serum IL-10 levels of rats in each group; **(Ci,Ciii)**: The MPO activity within rats’ organs in each group; **(Di,Diii)**: The levels of MDA within rats’ organs in each group. The results are presented as the mean ± SEM. **p < 0.01, ***p < 0.005, ****p < 0.0001 vs. sham group, ^#^ *p <* 0.05, ^###^ *p <* 0.005, ^####^ *p <* 0.0001 vs. SAP group; ns, *p >* 0.05. sham: sham operation group; SAP: Sever acute pancreatitis group; DCP: Dao-Chi powder treatment group.

As shown in Figure 4A, SAP caused an elevation in serum IL-6 levels (*p <* 0.01). Moreover, DCP administration significantly increased serum IL-10 levels (*p <* 0.005) and slightly decreased serum IL-6 levels (Figures 4A,B). Meanwhile, the activity of MPO in the duodenum, colon, and heart of rats in the SAP group was significantly enhanced compared to that in the sham group (all *p <* 0.001; Figures 4Ci,Ciii), which was significantly inhibited following DCP intervention (*p <* 0.001, *p <* 0.005, *p <* 0.005, respectively; Figures 4Ci,Ciii). In addition, SAP caused an increase in MDA levels in the three tissues (*p <* 0.005, *p <* 0.01, and *p <* 0.01, respectively; Figures 4Di,Diii). Conversely, DCP treatment effectively reversed SAP-induced up-regulation of MDA in the duodenum (*p <* 0.01; Figure 4Di) and slightly lowered MDA levels in the colon and heart (Figures 4Dii,Diii). Taken together, SAP caused neutrophil infiltration and oxidative stress in the duodenum, colon, and cardiac tissues, and DCP suppressed inflammation within these tissues and remarkably reduced oxidative stress in the duodenum.

### Dao-Chi Powder Regulated the Nuclear Factor Erythroid 2-Related Factor 2/Heme oxygenase-1/High Mobility Group Box-1 Signaling Pathways in Intestinal and Cardiac Tissues of Rats With Severe AP

**FIGURE 5:**
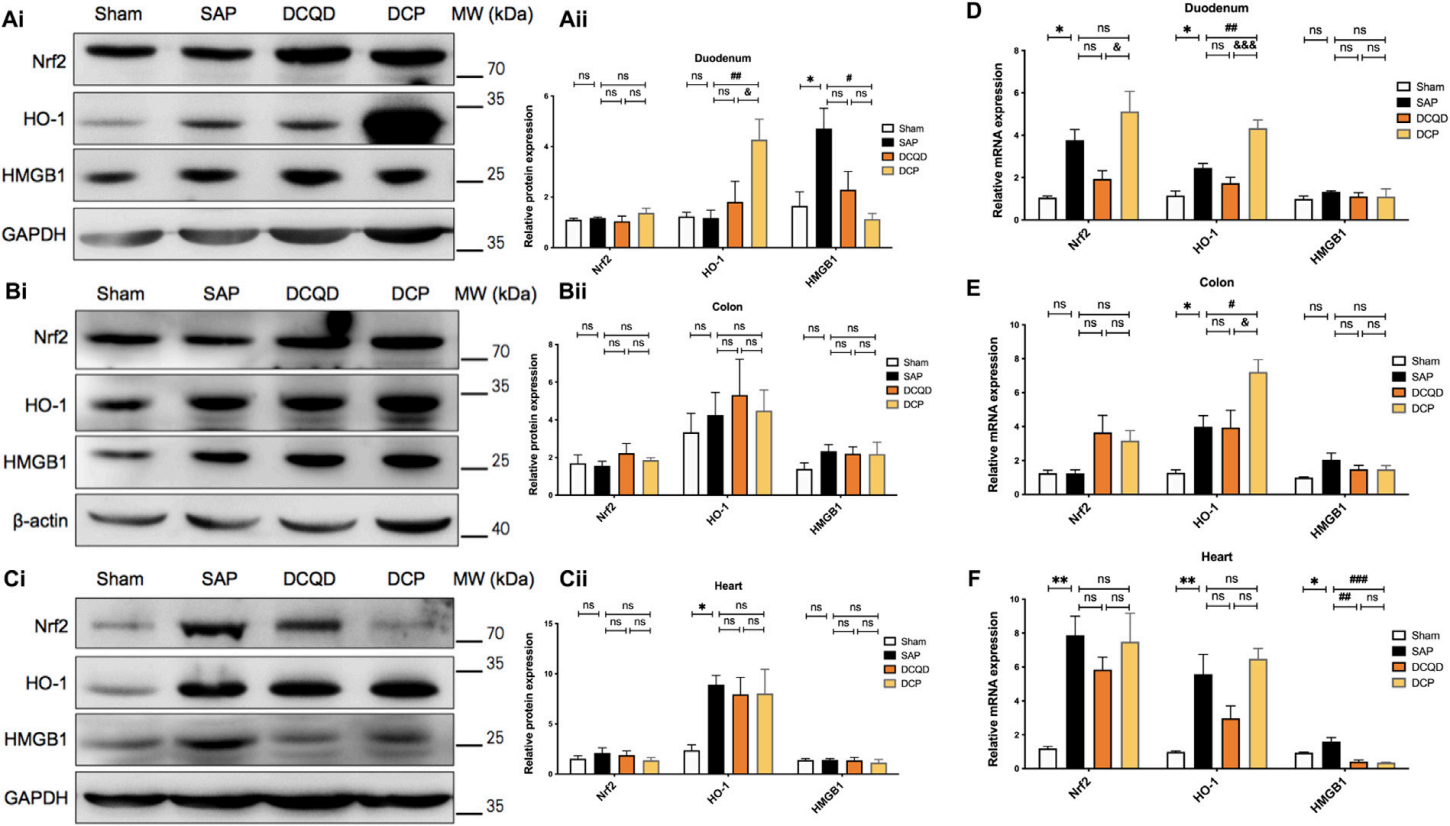
DCP regulated the Nrf2/HO-1/HMGB1 signaling pathways in intestinal and cardiac tissues of rats with SAP. **(A–C)**: Western blot analysis of Nrf2, HO-1 and HMGB1 protein expression in the duodenum, colon, and heart **(Ai,Ci)** and corresponding grayscale statistics **(Aii,Cii)**. Organs from rats in DCQD (9.6 mg/kg.BW) treatment group was included as a control. lane 1, sham; lane 2, SAP; lane 3, DCQD (control); lane 4, DCP. **(D–F)**: RT-PCR detection of Nrf2, HO-1, and HMGB1 mRNA expression in the rats’ organs in each group. The results are presented as the mean ± SEM. *p < 0.05, **p < 0.01 vs. sham group, ^#^ *p <* 0.05, ^##^ *p <* 0.01, ^###^ *p <* 0.005 vs. SAP group; ^&^ *p <* 0.05, ^&&&^ *p <* 0.005 vs. DCQD group; ns, *p >* 0.05. sham: sham operation group; SAP: Sever acute pancreatitis group; DCQD: Da-Cheng-Qi decoction treatment group; DCP: Dao-Chi powder treatment group.

We further explored the mechanism by which DCP regulated oxidative stress and inflammation. Da-Cheng-Qi decoction (DCQD) has been proved to be one of the most commonly used Chinese herbal prescriptions with anti-inflammatory effects on rats with SAP (Zhao et al., 2015; Yao et al., 2020). Accordingly, the tissues from DCQD-treated rats were used as a control for further exploration of the mechanism (detailed information provided in Supplementary Material, Supplementary method). As detected by Western blot, compared with the sham group, no significant changes were observed in the protein expression of Nrf2 in the duodenum, colon, and heart of rats with SAP (Figures 5A–C). In addition, the expression of HMGB1 in the duodenum and HO-1 in the heart of rats with SAP was much higher than that in the sham group (both *p <* 0.05; Figures 5Ai,Aii,Ci,Cii). Compared to the SAP group, Nrf2 expression in the duodenum displayed an upward trend in the DCP group (Figures 5Ai,Aii). Furthermore, after DCP administration, the expression of HO-1 in the duodenum significantly increased (*p <* 0.01; Figures 5Ai,Aii), while the expression of HMGB1 in the duodenum significantly decreased (*p <* 0.05; Figures 5Ai,Aii). Additionally, the expression of HO-1 in the duodenum in the DCP group was much higher than that in the DCQD group (*p <* 0.05; Figures 5Ai,Aii). The protein expression levels of Nrf2, HO-1, and HMGB1 in the duodenum, colon, and heart in the DCQD group were not significantly different from those in the SAP group (Figures 5A–C).

Moreover, as shown in Figures 5D–F, compared with the sham group, the mRNA levels in the SAP group were significantly increased, including Nrf2/HO-1 in the duodenum (both *p <* 0.05; Figure 5D), HO-1 in the colon (*p <* 0.05; Figure 5E), and Nrf2/HO-1/HMGB1 in the heart (*p <* 0.01, *p <* 0.01, and *p <* 0.05, respectively; Figure 5F). Compared with the SAP group, the mRNA level of Nrf2 in the duodenum and colon displayed an upward trend in rats in the DCP group (Figures 5D,E). Notably, DCP administration significantly increased the mRNA levels of HO-1 in the duodenum and colon (*p <* 0.01, *p <* 0.05, respectively; Figures 5D,E). Meanwhile, DCP and DCQD intervention significantly decreased the mRNA level of HMGB1 in the heart (*p <* 0.005, *p <* 0.01; Figure 5F). In addition, the DCP treatment group showed higher mRNA levels of Nrf2 and HO-1 in the duodenum and higher HO-1 levels in the colon than those in the DCQD group (*p <* 0.05, *p <* 0.005, *p <* 0.05, respectively; Figures 5D,E). Taken together, DCP could activate the Nrf2/HO-1 pathway in the duodenum and colon tissues of rats with SAP and inhibit the expression of HMGB1 in the duodenum and heart.

### Comparison of the Active Components of Dao-Chi Powder Targeting to Intestinal and Cardiac Tissues of Severe AP Rats

To compare the differential tissue distributions of DCP components in the sham and SAP groups, we used HPLC-MS/MS technology. The data showed that ten components of the DCP were accurately quantified (data presented in Supplementary Figure S1). Six of the ten monomers could be detected in the duodenum, colon, and cardiac tissues. As shown in Table 4, compared with the sham group, the concentrations of ajugol and oleanolic acid in the duodenum in the SAP group were significantly higher (*p <* 0.01, *p <* 0.05), and the concentrations of ajugol in the colon and liquiritigenin in the heart were significantly lower (*p <* 0.01, *p <* 0.01). These results suggest that SAP might promote the distribution of ajugol and oleanolic acid to the duodenum, whereas it inhibits the distribution of liquiritigenin to the heart and ajugol to the colon, suggesting that the duodenum might be the main target organ of DCP in rats with SAP.

**TABLE 4:** Major components of Dao-Chi powder detected by HPLC-MS/MS.

Monomers	Group	Duodenum (ng/ml)	Colon (ng/ml)	Heart (ng/ml)
Ajugol	sham	5.13±0.66	103.90±22.89	N.D.
	SAP	11.02±1.48**	10.64±3.91**	N.D.
Oleanolic acid	sham	0.31±0.14	1.32±0.36	N.D.
	SAP	1.11±0.28*	2.24±1.10	N.D.
Stigmasterol	sham	36.51±8.01	N.D.	N.D.
	SAP	43.66±5.98	N.D.	N.D.
Chlorogenic acid	sham	64.55±13.84	45.25±2.82	67.72±7.33
	SAP	83.85±28.12	38.17±7.08	37.64±12.16
Glycyrrhizic acid	sham	4.56±1.02	5.24±1.41	2.09±0.26
	SAP	7.37±1.12	3.93±1.22	2.33±0.41
Liquiritigenin	sham	1.62±0.66	1.57±0.46	0.26±0.05
	SAP	1.66±0.62	1.15±0.38	0.04±0.02**

Sham, sham operation group; SAP, Sever acute pancreatitis group; N.D., Not detected; *p < 0.05, **p < 0.01 vs. sham.

### Interactions and Potential Binding Modes of Dao-Chi Powder Components and the Targets Nrf2, Heme oxygenase-1 and High Mobility Group Box-1

**FIGURE 6:**
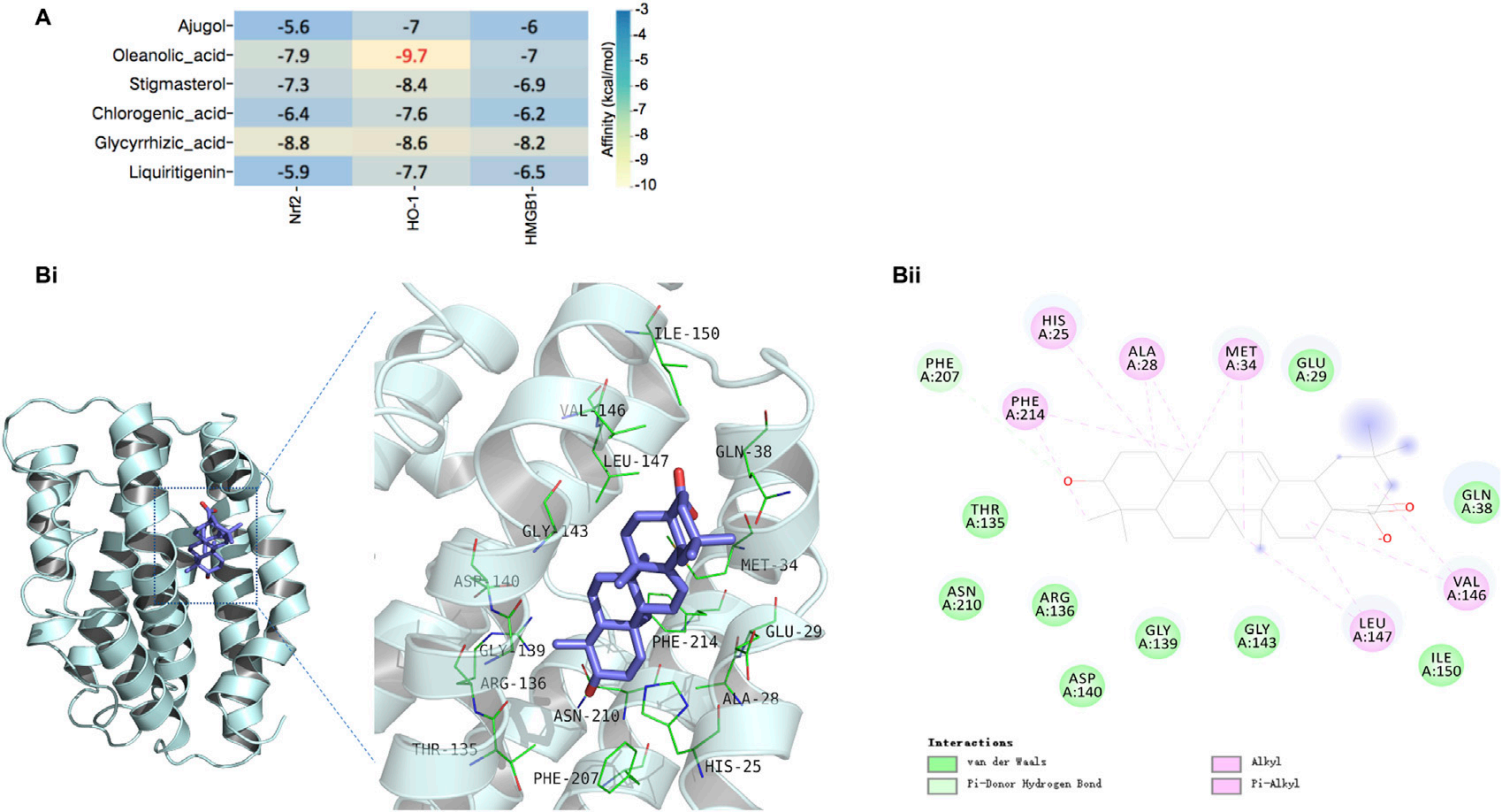
Interactions and potential binding modes of DCP components and the targets Nrf2, HO-1 and HMGB1. **(A)**: The docking score of the detected DCP components in rat tissues with the targets of Nrf2, HO-1 and HMGB1. **(Bi,Bii)**: Representative 3D binding model of oleanolic acid to HO-1(Bi), and representative 2D model of the binding (Bii).

To explore the potential interactions and binding modes between the detected DCP components and their targets (Nrf2, HO-1, and HMGB1), a molecular docking analysis was performed. The docking scores of the six key components of DCP and the three targets are presented in Figure 6A, which suggests that all detected DCP compounds had good binding affinities with Nrf2, HO-1, and HMGB1. Moreover, a lower free binding energy indicates a more stable binding conformation, and the molecules with the highest affinity for Nrf2, HO-1, and HMGB1 are glycyrrhizic acid, oleanolic acid, and glycyrrhizic acid, respectively. The highest binding affinity was observed between the oleanolic acid molecule and the target HO-1, and the representative visualized molecular binding models are shown in Figure 6Bi-Bii.

## Discussion

In the present study, NaT-induced SAP caused oxidative stress lesions and inflammatory responses in the duodenum, colon, and cardiac tissues of rats, which could be suppressed by DCP. Mechanistically, we found that DCP protected against SAP-related intestinal and cardiac injuries by balancing the anti-/pro-inflammatory cytokines, inhibiting the accumulation of neutrophils, and oxidative stress partially through activation of the Nrf2/HO-1 pathway and inhibition of HMGB1. Moreover, we identified the potential protective components of DCP using HPLC-MS/MS analysis and verified its binding ability using molecular docking analysis.

During AP, regardless of the initiating factor, damage to acinar cells is accompanied by the release of various inflammatory mediators that first activate local inflammatory reactions. Without full control over time, the inflammatory cascade may eventually cause multiple organ dysfunctions (Dikmen et al., 2018). Thus, blocking inflammatory cascade amplification plays a decisive role in the prognosis of SAP (Dawra et al., 2011). DCP has attracted extensive attention in Chinese research, and its powerful anti-inflammatory and antioxidant properties have been therapeutically validated in a variety of inflammatory diseases such as viral myocarditis and hand-foot-mouth disease in the clinic (Zhou and Xue, 1998; Yang et al., 2019). In this study, we demonstrated the protective effects of DCP on NaT-induced SAP in rats, as indicated by the decrease in serum amylase levels and pancreatic pathological scores.

SAP is often accompanied by imbalance of gastrointestinal hormones, including the up-regulation of VIP (inhibiting gastrointestinal motility) and down-regulation of MTL (promoting gastrointestinal motility), resulting in gastrointestinal motility disorder (Wang et al., 2003). In addition, intestinal barrier damage and permeability changes can lead to intestinal microbiota and/or ET translocation, manifested by significantly up-regulated ET and D-Lac in the peripheral blood (Zhongkai et al., 2012). Consistent with previous studies, SAP up-regulated the serum levels of ET, D-Lac, and VIP in this study, and the current experiment innovatively confirmed that DCP administration directly inhibited these factors and slightly up-regulated the serum MTL levels. Additionally, we found that serum levels of cTnI and CK-MB, common markers used in assessing cardiac function, were both decreased after DCP intervention compared to those in the SAP group. Combined with further histopathological examination, it was confirmed that DCP could alleviate not only the pathological injury of the pancreas itself but also the injuries of the intestines and heart in rats with SAP.

After the onset of AP, damaged acinar cells and activated immune cells release diverse inflammation-associated cytokines including TNF-α, IL-6, and IL-10 (Chen et al., 2021). MPO, a peroxidase enriched in neutrophils, is a common indicator of tissue neutrophil immersion, and is involved in mediating tissue inflammation and oxidative stress (Zhongkai et al., 2012). It is the most frequently measured product of membrane lipid peroxidation. MDA can not only cause structural damage to biofilms but can also combine with intracellular proteins or nucleic acids to affect a series of pathophysiological processes in the body and can be used as a common indicator to reflect the degree of tissue oxidative stress damage (Chen et al., 2021). Considering that pharmacologic intervention against oxidative stress damage might be beneficial in delaying onset or progression of several diseases (Drake et al., 2003), we also compared the MDA levels in this study. consistent with previous studies (Yang et al., 2022), in our present study, SAP induced a significant elevation of MPO activity and MDA levels in experimental rats. Importantly, DCP treatment restored the pro-/anti-inflammatory balance, decreased MPO activity in the duodenum, colon, and cardiac tissues, and particularly increased MDA levels in the duodenum of rats with SAP, revealing a regulatory role of DCP in inflammatory responses and oxidative stress in Nat-induced SAP rats.

To further explore the upstream mechanism of DCP regulation of oxidative stress and inflammation, we focused on Nrf2, a ubiquitous transcription factor involved in the regulation of redox state by the induction of downstream redox-dependent genes, called vitagenes (such as HO-1), which sense redox perturbations and actively operate in promoting cell survival under various stimuli including oxidative stress (Mancuso et al., 2006; Miquel et al., 2018). Moreover, in numerous experimental models, natural antioxidants have been shown to induce hormetic dose responses that are mediated *via* the activation Nrf2 and related antioxidant response elements, meanwhile, these antioxidant activators (inducers) of the Nrf2/HO-1 pathway also has anti-inflammatory activity. Interestingly, the intracellular protective effects of these natural antioxidants through hormesis were evident only in wild-type but not in Nrf2-knockout mice. (Calabrese et al., 2010). Furthermore, another study showed that higenamine reduced HMGB1 expression in IR injury-performed intestines, which was inhibited by additional administration of an HO-1 inhibitor, indicating that Nrf2/HO-1 activation is indeed involved in the reduction of HMGB1 production. In addition, recent studies have shown that isoproterenol can inhibit the release of HMGB1 and improve the survival rate of mice with sepsis by mediating HO-1 induction through Nrf2 translocation (Ha et al., 2011). Accumulating evidence has shown that the Nrf2/HO-1 pathway is involved in the inflammatory response to AP. Yang et al. reported that triptolide exerted a therapeutic role in cerulein-induced AP by activating the expression of Nrf2 and HO-1 *in vivo* and *in vitro* (Yang et al., 2022). Liu et al. found that antibody neutralization of HMGB1 decreased the intestinal injury score, MPO activity, and levels of TNF-α and IL-6 in an intestinal I/R injury mouse model. Furthermore, they confirmed that activation of Nrf2/HO-1 resulted in the inhibition of HMGB1 expression, whereas pharmacological inhibition of HO-1 or transfection with Nrf2 siRNA restored the expression of HMGB1 (Liu et al., 2015). In addition, in the same NaT-induced SAP model, Nrf2/HO-1 inhibited NLRP3 inflammasome activation and further mitigated SAP-associated lung injury (Gao et al., 2020). However, whether DCP affects Nrf2/HO-1/HMGB1 signaling in SAP remains unknown. DCQD has been proven to be one of the most commonly used Chinese herbal prescriptions with anti-inflammatory effects in the treatment of AP, and earlier studies by our team have also confirmed that DCQD could effectively alleviate multi-organ inflammatory responses in rats with SAP (Zhao et al., 2015; Yao et al., 2020). Moreover, DCQD-derived compounds could reduce the levels of serum cTn1 and CK-MB, reduce the apoptosis of myocardial cells, and mitigate the pathological damage of pancreatic and cardiac tissues in rats with SAP (Li et al., 2015). In the mechanism exploration section, DCQD was selected as the control. Western blot and RT-PCR results confirmed that DCP treatment enhanced the expression of Nrf2 in the duodenum and activated the gene encoding and protein expression of HO-1 in the duodenum and colon, suggesting that DCP has a regulatory effect on the oxidative stress response in the duodenum and colon of rats with SAP. Moreover, DCP intervention significantly down-regulated the expression of HMGB1 in the duodenum and heart, further verifying its anti-inflammatory properties. Additionally, our data showed that one-time DCQD gavage had limited regulatory effects on the Nrf2/HO-1 pathway within the organ tissues of rats with SAP but significantly down-regulated the expression of HMGB1 in the heart, suggesting the anti-inflammatory potential of DCQD in the heart of rats with SAP, which is worthy of further dose-effect verification experiments.

Next, to speculate on the material basis of DCP alleviating oxidative stress and inflammation, the tissue distributions of DCP components in the duodenum, colon, and heart were determined by HPLC-MS/MS, and six components were accurately quantified. Among them, the concentrations of ajugol and oleanolic acid in the duodenum of rats with SAP were higher than those in the sham group, which is consistent with tissue pharmacology theory (Zhao et al., 2015). In other words, the specific monomer components of Chinese medicine are absorbed into the blood and then targeted to pathological organs to play a pharmacological role. Accordingly, we speculated that ajugol and oleanolic acid might be the most promising components responsible for the protective effects of DCP on the duodenum during SAP. Impaired autophagy is a cellular event central to AP pathogenesis (Lee and Papachristou, 2019). Ajugol has been shown to promote lysosomal biogenesis, facilitating fusion of autophagosomes and lysosomes, thereby leading to an improvement in autophagy flux (Zhang et al., 2021). Similarly, oleanolic acid alleviates hepatic I/R injury by reducing HMGB1 release and regulating autophagy flux (Wang et al., 2019). Besides, oleanolic acid has been reported to be a natural agonist of Nrf2 antioxidant pathway (Tsai and Yin, 2008). Du and Ko (2006) reported that oleanolic acid could protect against cardiac injury *via* regulating the glutathione-mediated mitochondrial antioxidant mechanism in a myocardial I/R rat model (Du and Ko, 2006). Castellano et al. demonstrated that oleanolic acid could inhibit the release of inflammatory factors (IL-1β, TNF-α, IL-6, etc.) under various inflammation and oxidative stress scenarios (Castellano et al., 2022). The molecular mechanisms mentioned above are all involved in AP, which might help decipher why DCP works better at regulating oxidative stress and inflammation in the duodenum. Moreover, SAP inhibited the distribution of ajugol in the colon and liquiritigenin in the heart, suggesting that SAP may affect the affinity and metabolic pattern of DCP in the colon and heart of rats.

Finally, we employed molecular docking analysis to further verify the interactions between the detected DCP components and the three targets of the Nrf2/HO-1/HMGB1 axis, which indicated that all six screened components of DCP had relatively good binding affinity with the target proteins (all ≤ −5 kcal/mol). It is worth noting that oleanolic acid, the component we hypothesized to have a therapeutic role in the duodenum of rats with SAP, had the highest binding affinity for HO-1, which may provide a basis for further efficacy verification and mechanistic exploration of oleanolic acid in the treatment of SAP.

## Conclusion

In summary, we confirmed that NaT-induced SAP in rats was complicated by pathological damage to the duodenum, colon, and heart, together with the imbalance of pro-/anti-inflammatory responses, inflammatory cell infiltration, and oxidative stress injury within these tissues. In addition, the Chinese herbal prescription DCP protected against intestinal and cardiac injuries by inhibiting oxidative stress and inflammation, at least partially, through regulating the Nrf2/HO-1/HMGB1 axis. Furthermore, based on HPLC-MS/MS and molecular docking analysis, we speculated that certain components (ajugol, oleanolic acid, etc.) of DCP might have a therapeutic effect on SAP. Thus, it may be important to explore the therapeutic potential of these monomers in SAP therapy and further clarify their mechanisms in future studies.

## Data Availability

The datasets presented in this study can be found in online repositories. The names of the repository/repositories and accession number(s) can be found in the article/Supplementary Material
