# Supplementary material for: Dao-Chi Powder Ameliorates Pancreatitis-Induced Intestinal and Cardiac Injuries via Regulating the Nrf2-HO-1-HMGB1 Signaling Pathway in Rats
Source: Front Pharmacol. 2022 Jul 11;13:922130. doi: 10.3389/fphar.2022.922130 (PMC9310041; doi:10.3389/fphar.2022.922130)
Supplement: Supplementary file 1 [file DataSheet2.ZIP › additional files (Raw data)/Raw data (Westerns blot).pptx]

## Slide 1
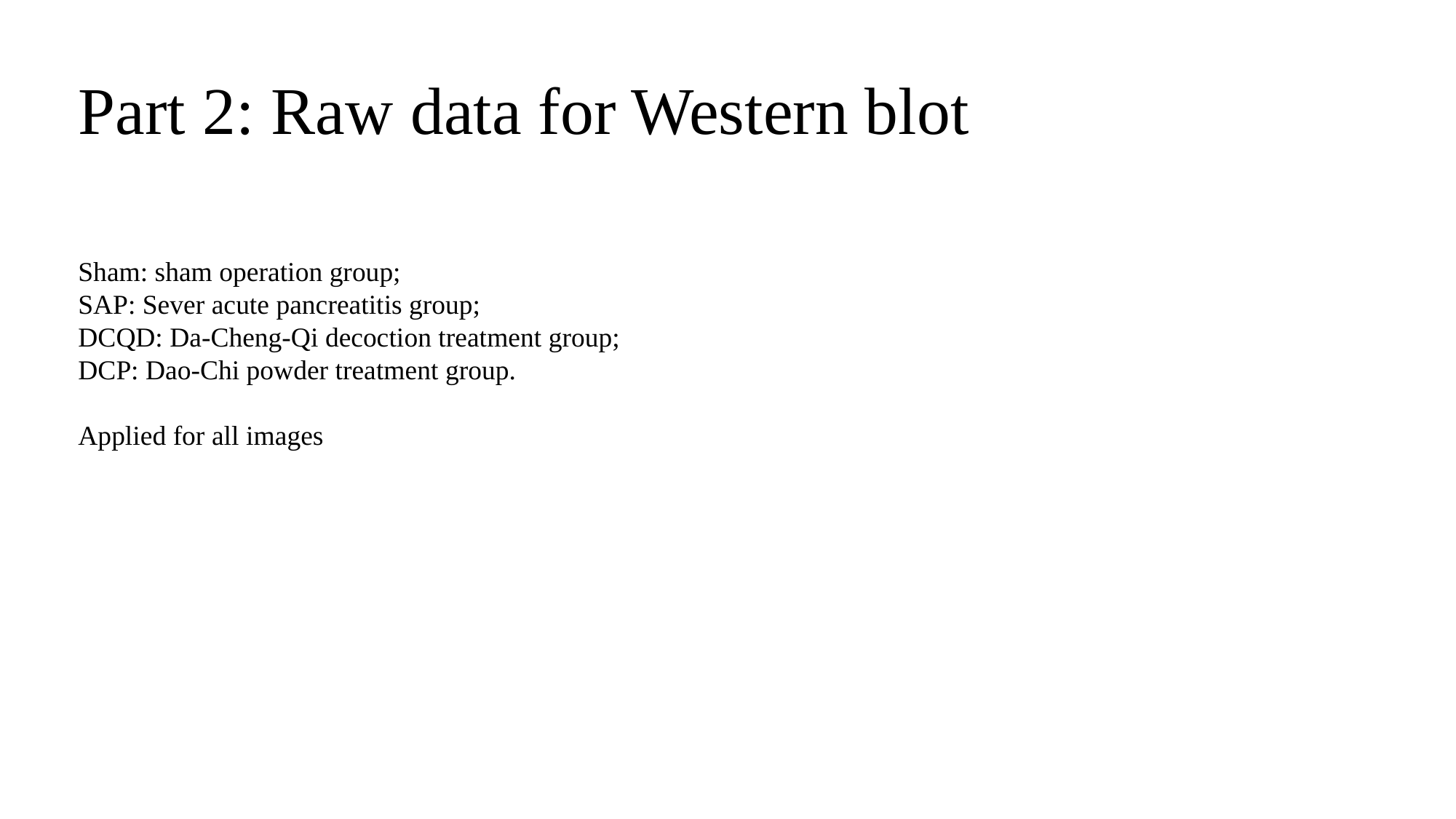

# Part 2: Raw data for Western blot
Sham: sham operation group;
SAP: Sever acute pancreatitis group;
DCQD: Da-Cheng-Qi decoction treatment group;
DCP: Dao-Chi powder treatment group.
Applied for all images

## Slide 2
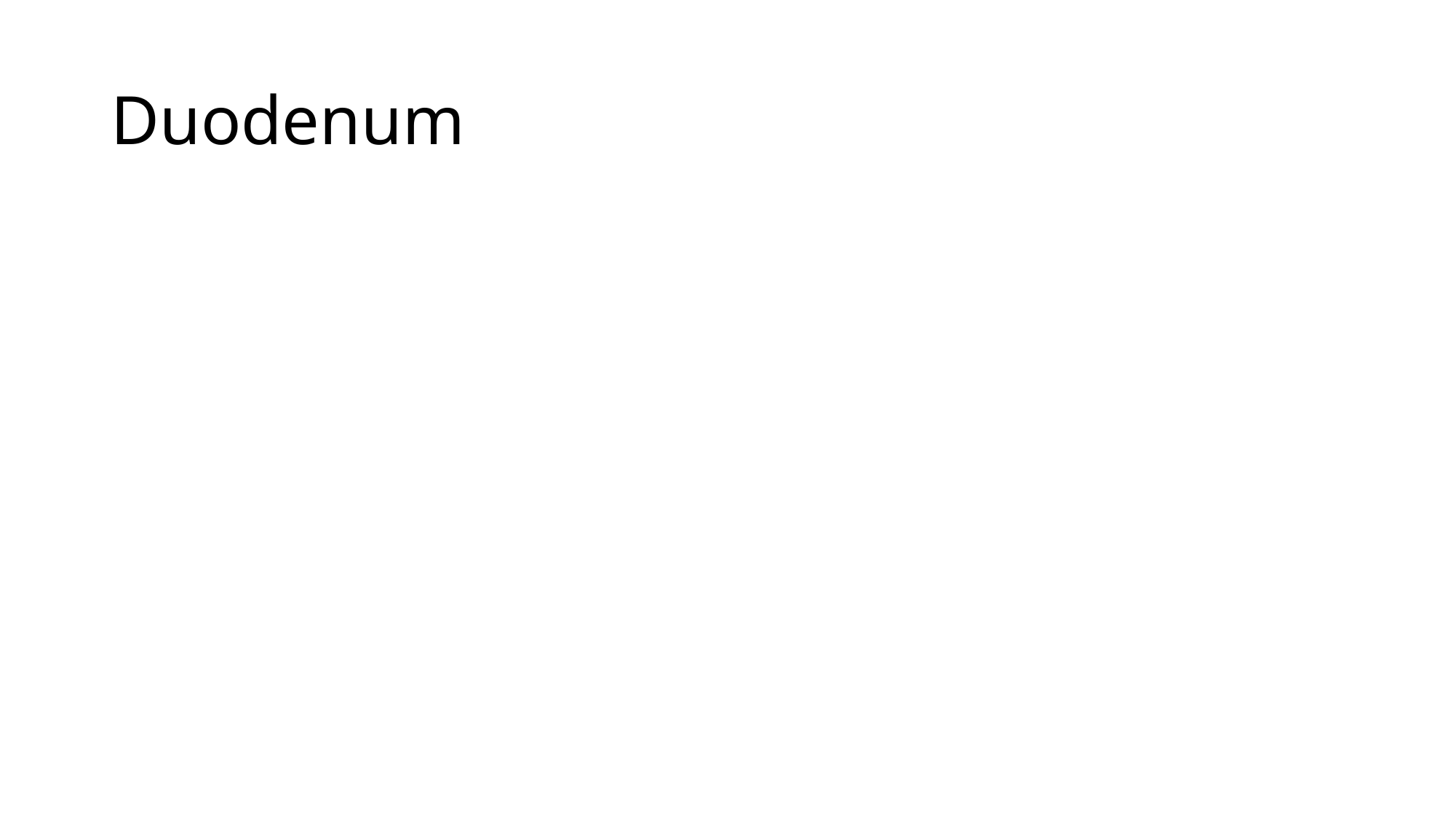

# Duodenum

## Slide 3
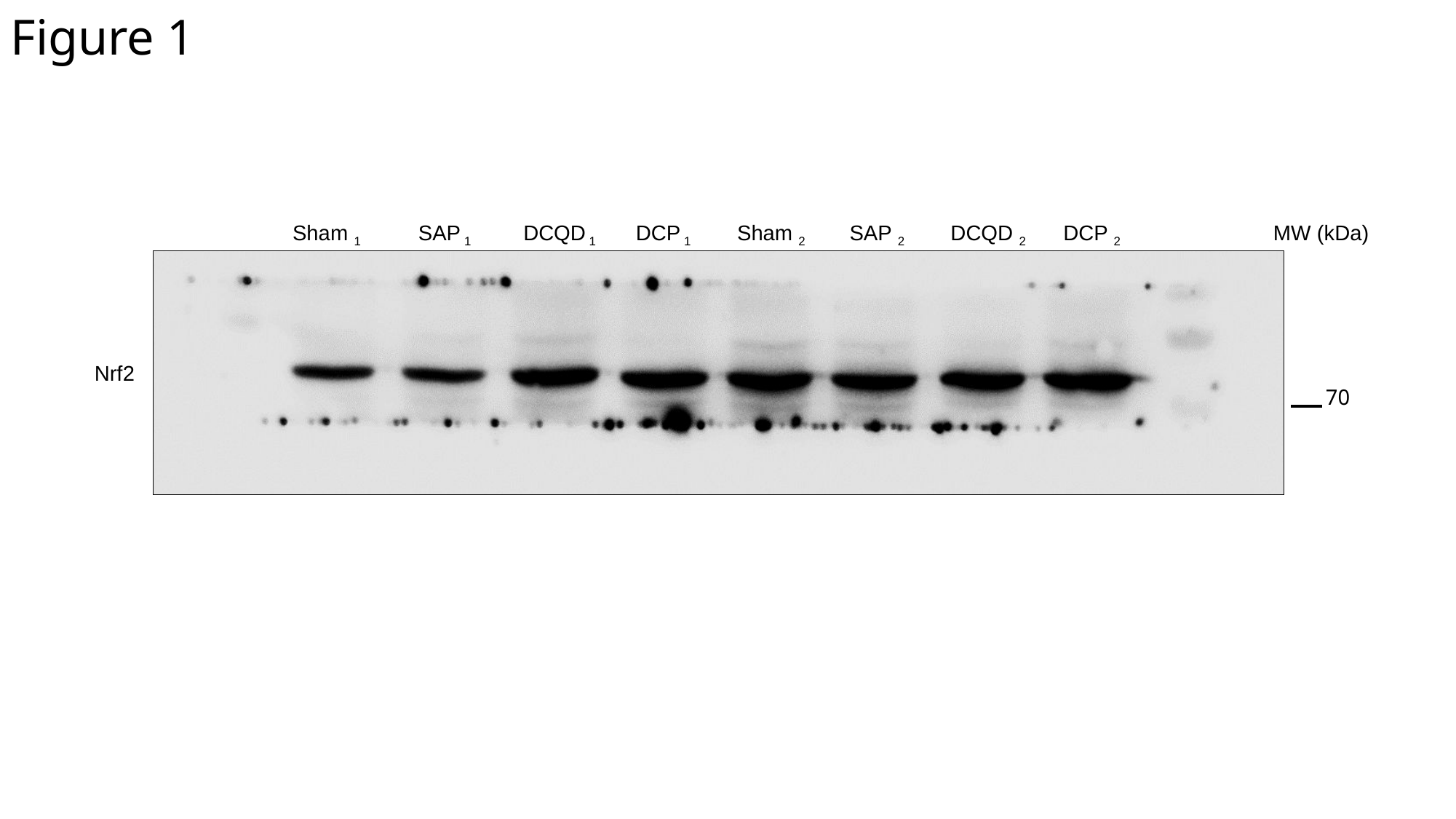

Figure 1
Sham 1
SAP 1
DCQD 1
DCP 1
Sham 2
SAP 2
DCQD 2
DCP 2
MW (kDa)
Nrf2
70

## Slide 4
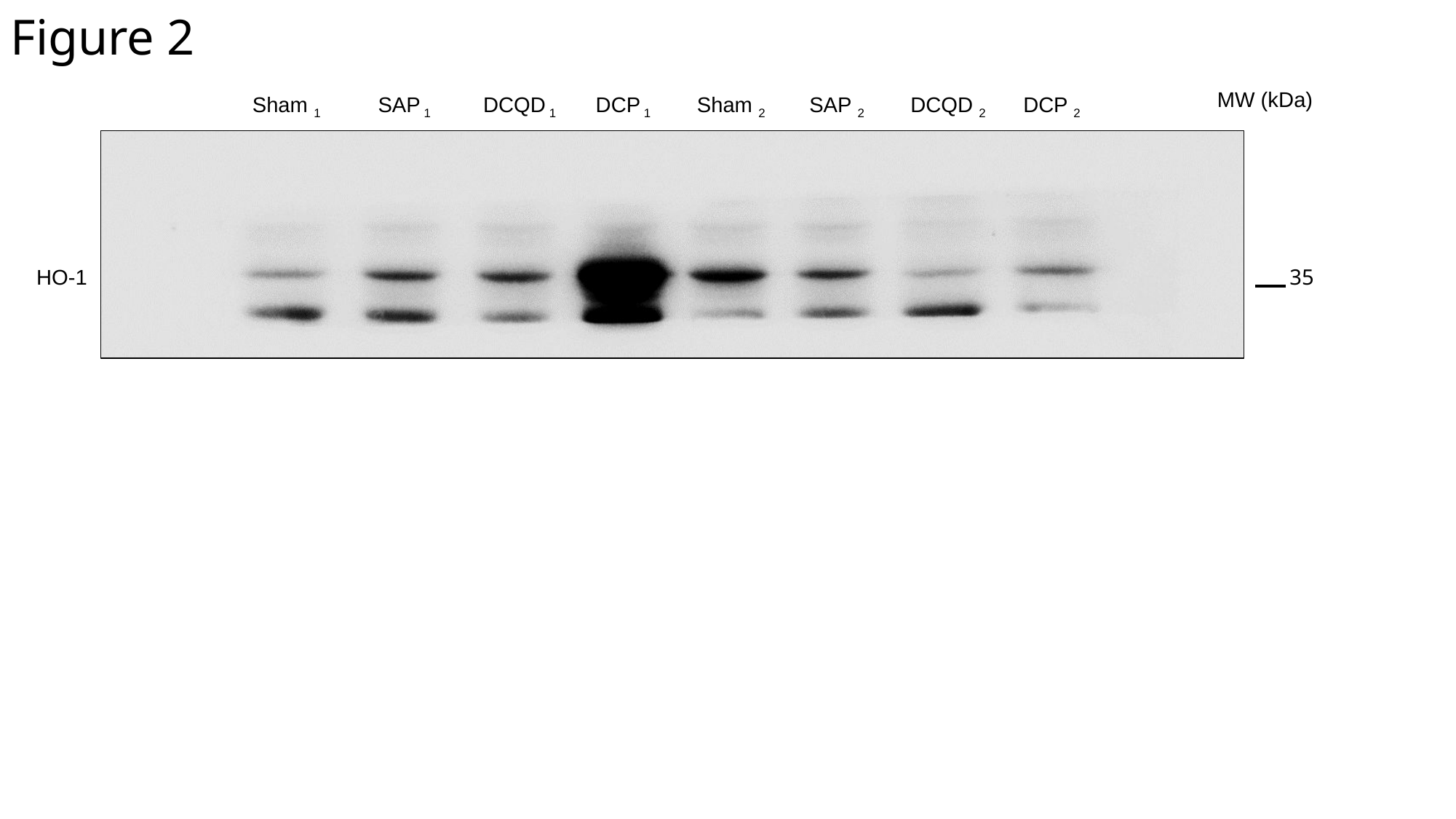

Figure 2
MW (kDa)
Sham 1
SAP 1
DCQD 1
DCP 1
Sham 2
SAP 2
DCQD 2
DCP 2
35
HO-1

## Slide 5
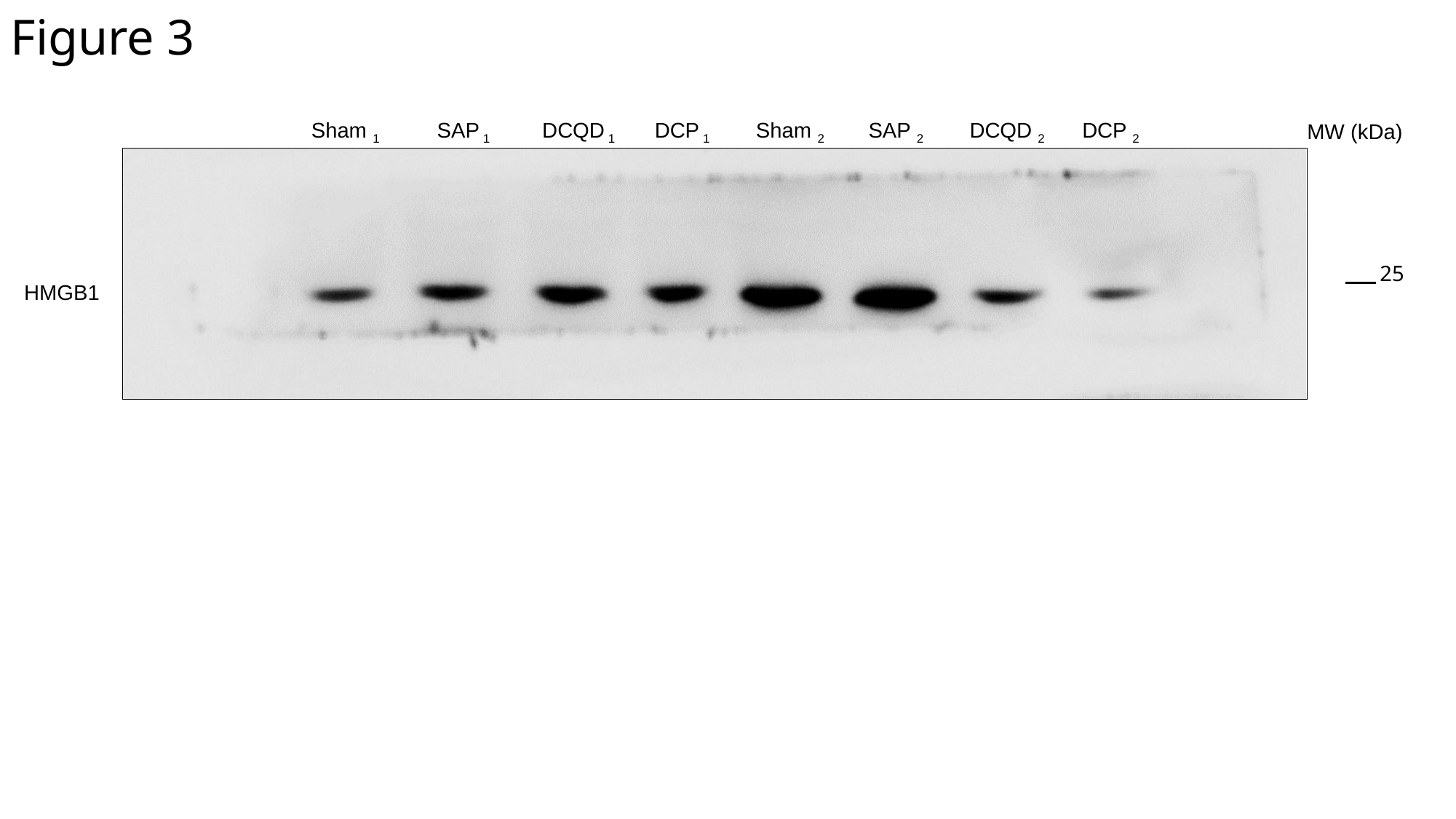

Figure 3
Sham 1
SAP 1
DCQD 1
DCP 1
Sham 2
SAP 2
DCQD 2
DCP 2
MW (kDa)
25
HMGB1

## Slide 6
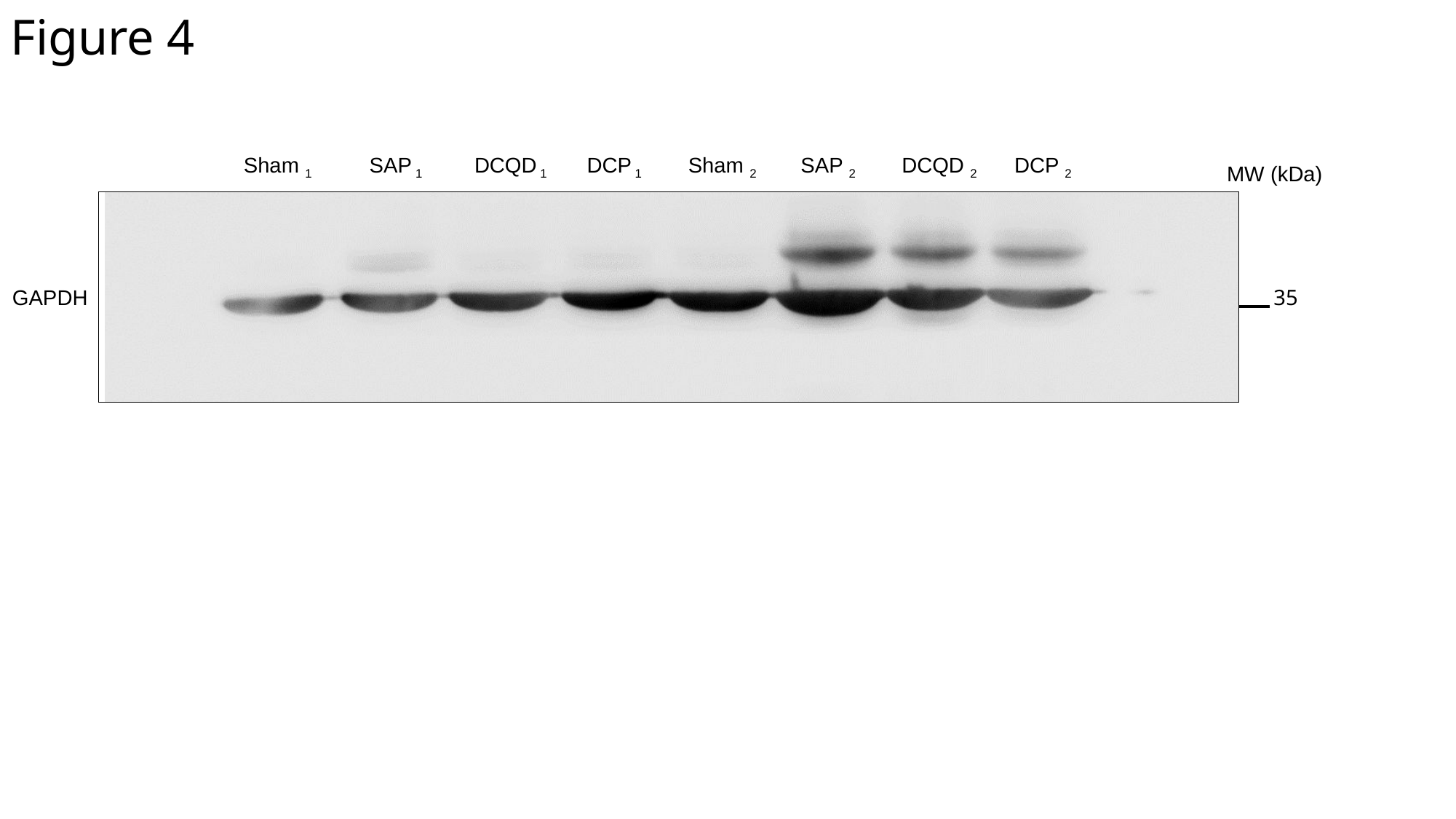

Figure 4
Sham 1
SAP 1
DCQD 1
DCP 1
Sham 2
SAP 2
DCQD 2
DCP 2
MW (kDa)
GAPDH
35

## Slide 7
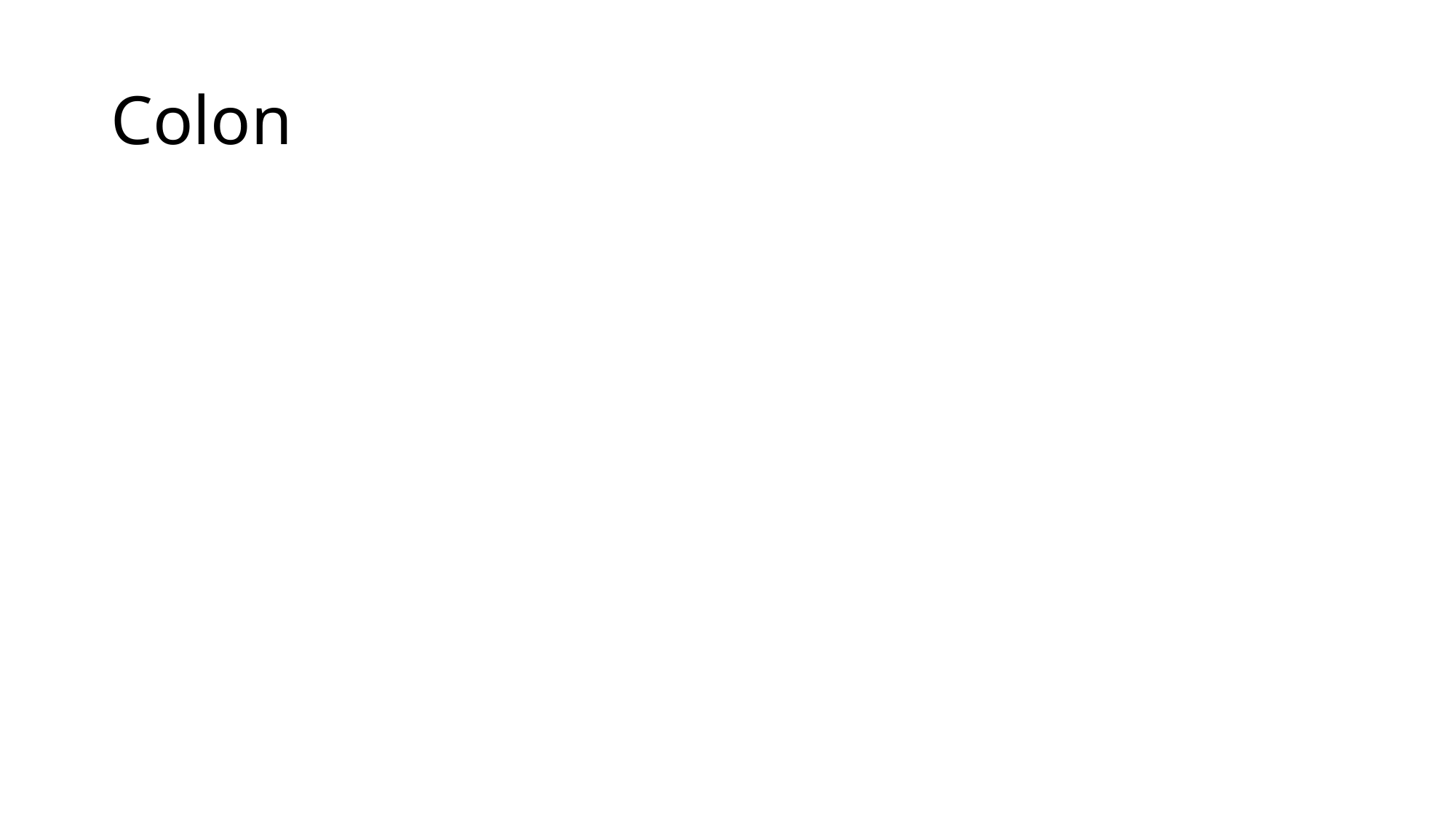

# Colon

## Slide 8
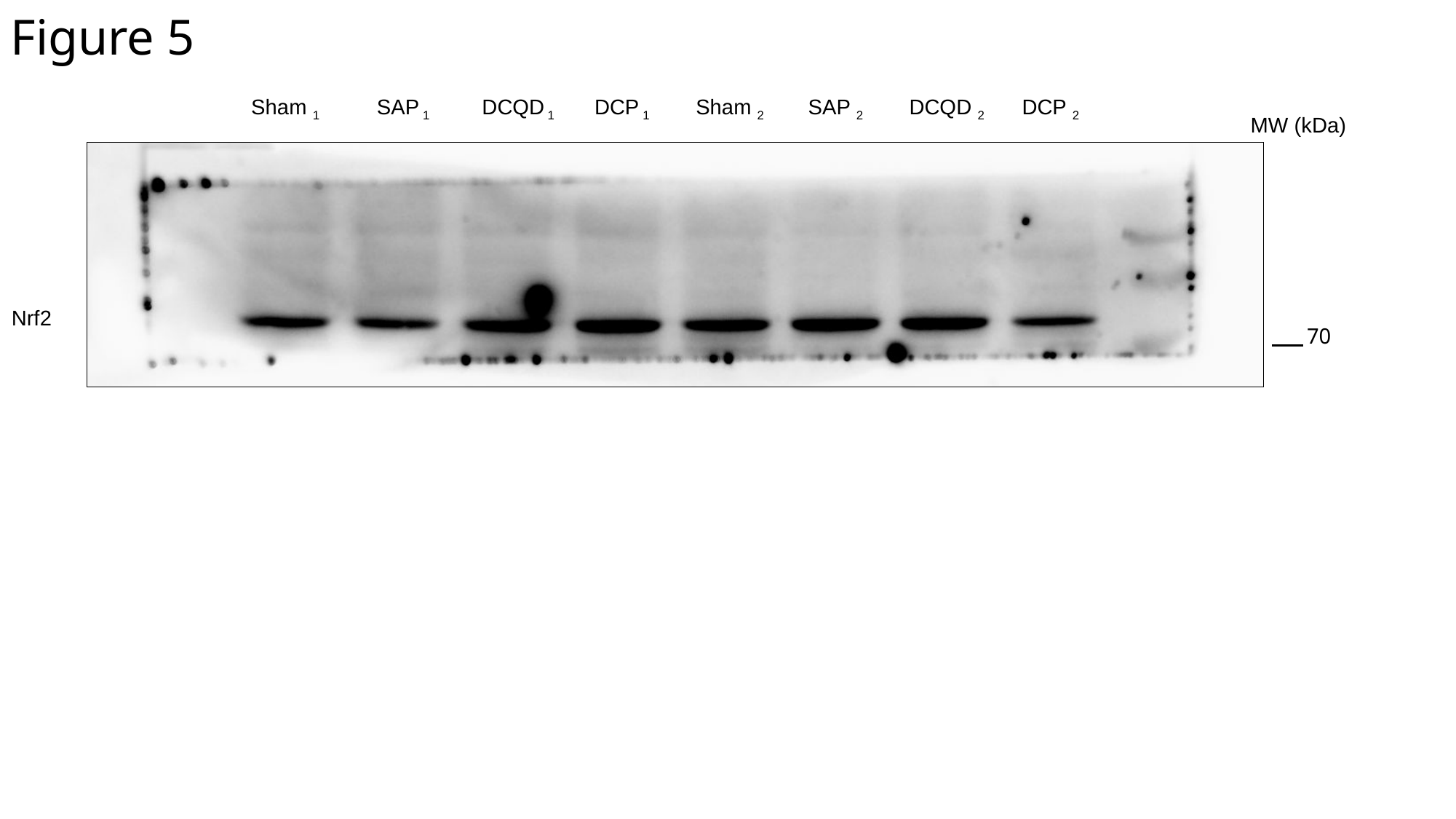

Figure 5
Sham 1
SAP 1
DCQD 1
DCP 1
Sham 2
SAP 2
DCQD 2
DCP 2
MW (kDa)
Nrf2
70

## Slide 9
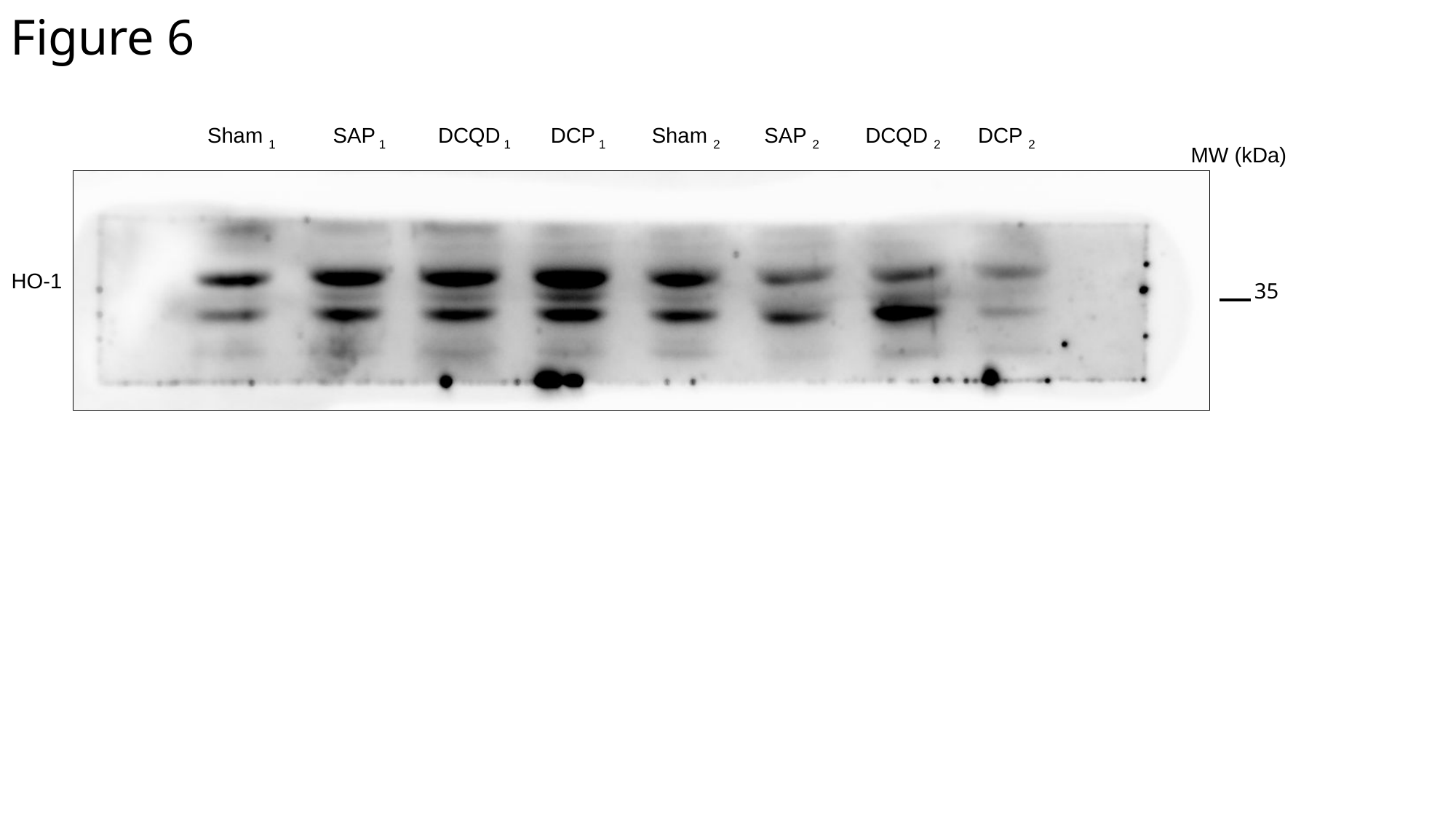

Figure 6
Sham 1
SAP 1
DCQD 1
DCP 1
Sham 2
SAP 2
DCQD 2
DCP 2
MW (kDa)
HO-1
35

## Slide 10
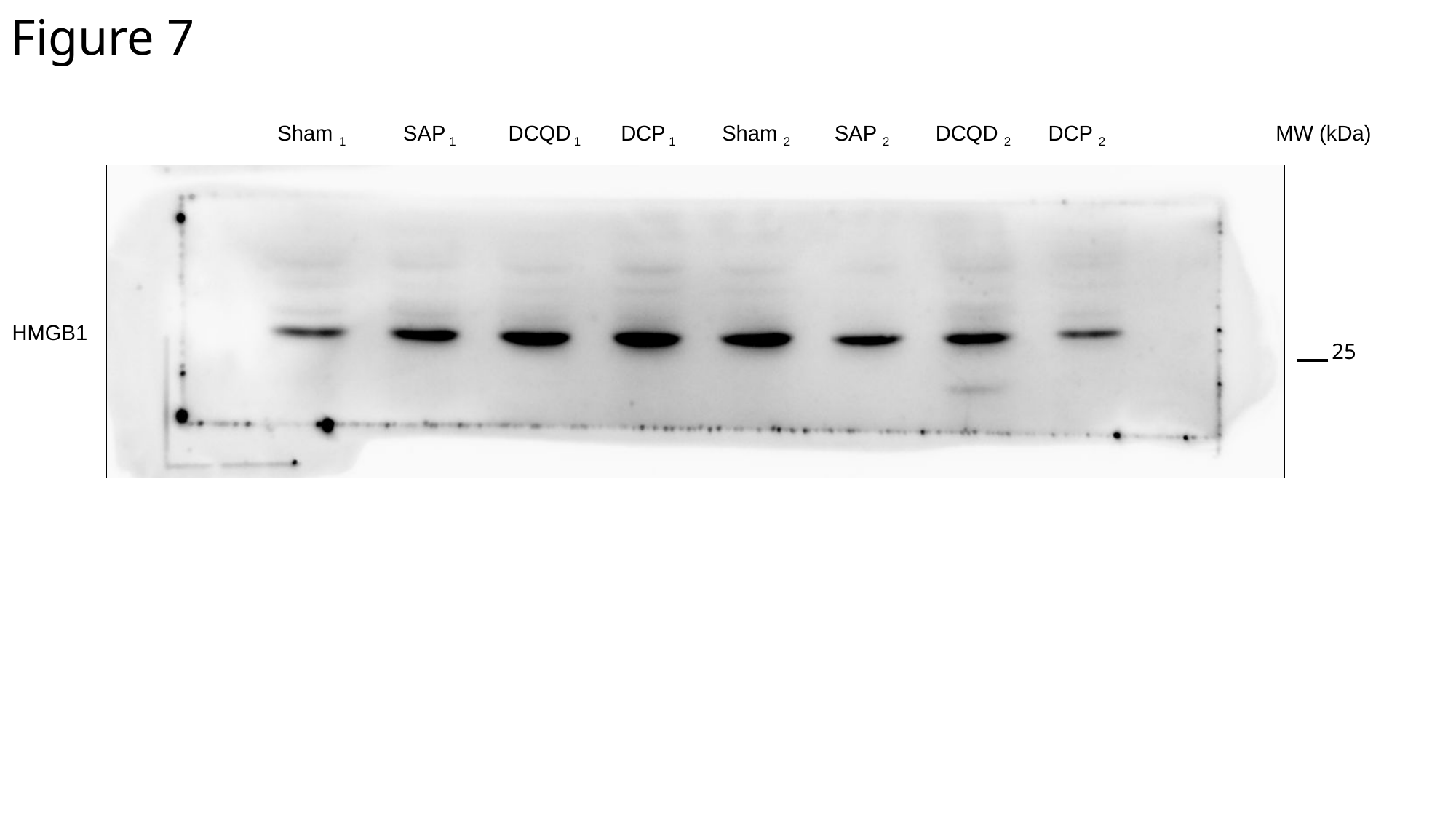

Figure 7
Sham 1
SAP 1
DCQD 1
DCP 1
Sham 2
SAP 2
DCQD 2
DCP 2
MW (kDa)
HMGB1
25

## Slide 11
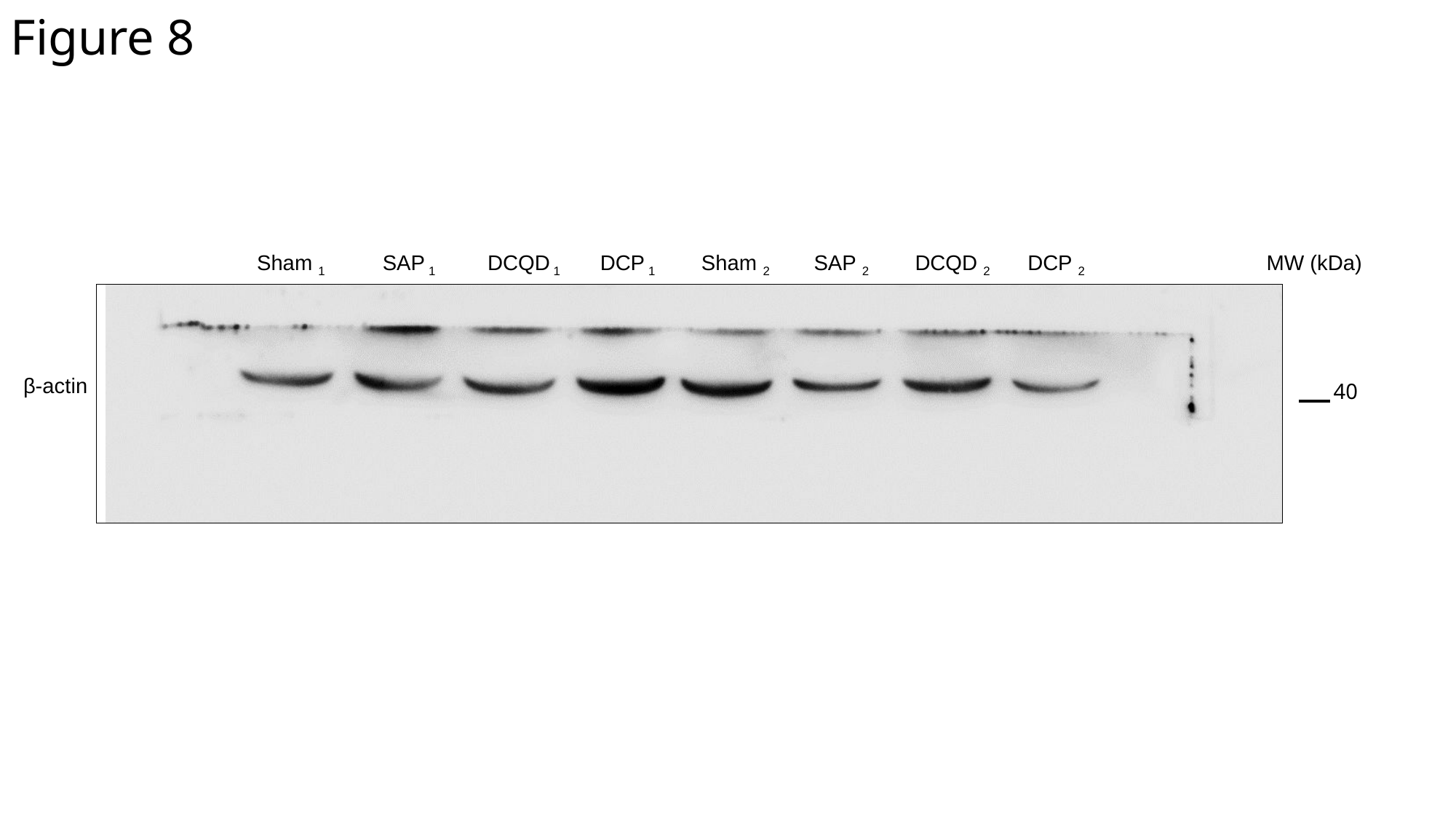

Figure 8
MW (kDa)
Sham 1
SAP 1
DCQD 1
DCP 1
Sham 2
SAP 2
DCQD 2
DCP 2
β-actin
40

## Slide 12
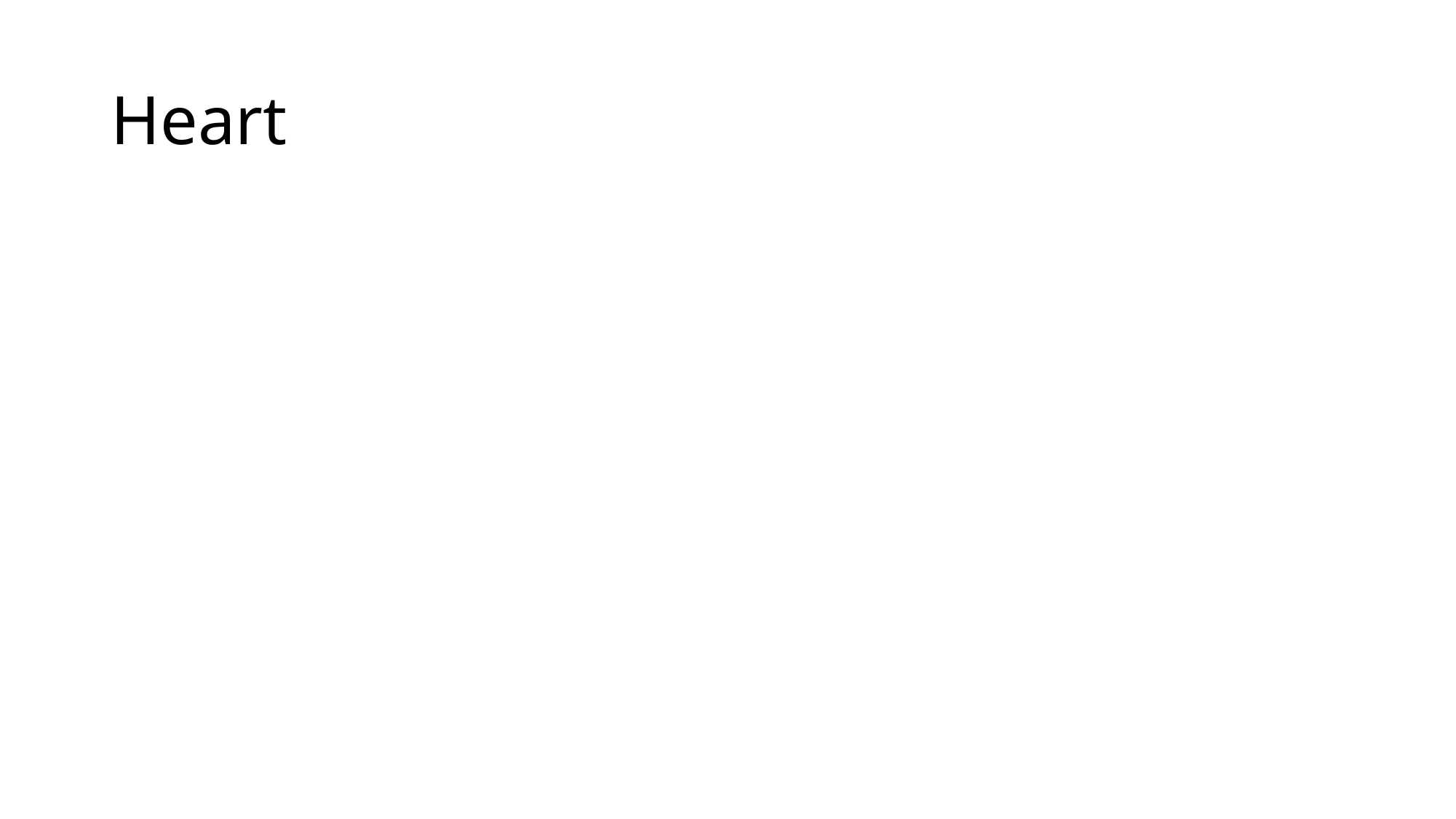

# Heart

## Slide 13
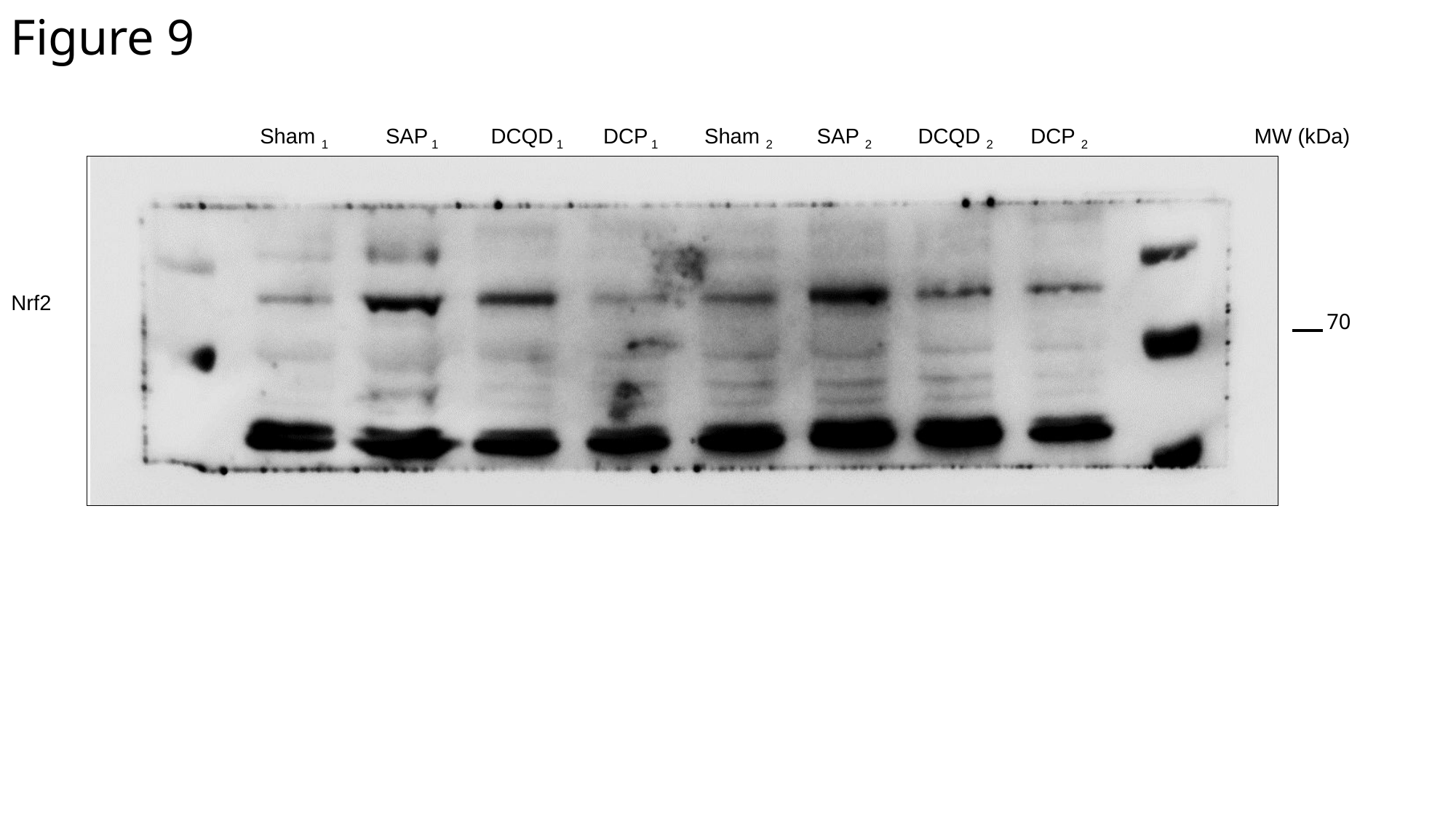

Figure 9
Sham 1
SAP 1
DCQD 1
DCP 1
Sham 2
SAP 2
DCQD 2
DCP 2
MW (kDa)
Nrf2
70

## Slide 14
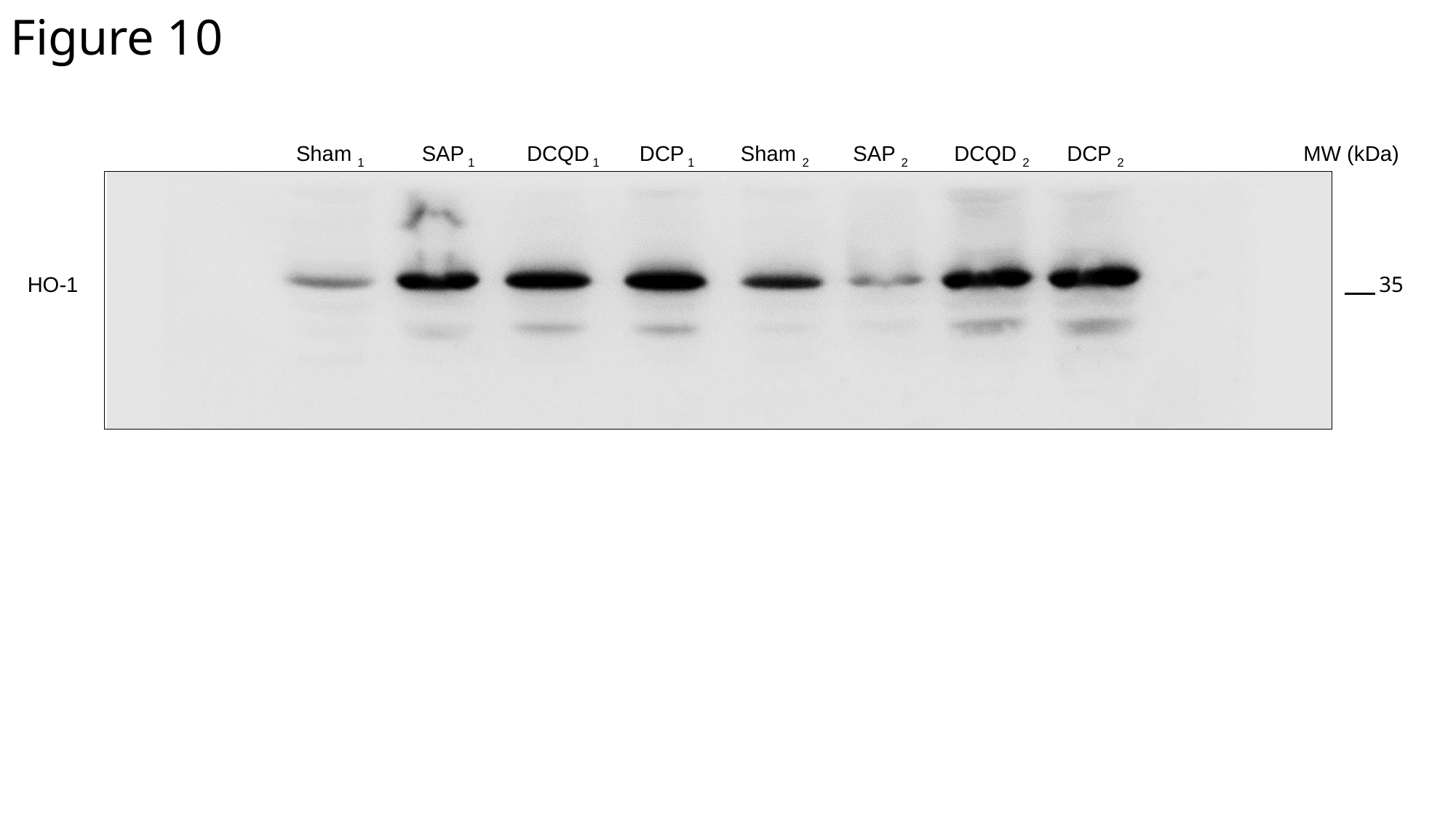

Figure 10
Sham 1
SAP 1
DCQD 1
DCP 1
Sham 2
SAP 2
DCQD 2
DCP 2
MW (kDa)
HO-1
35

## Slide 15
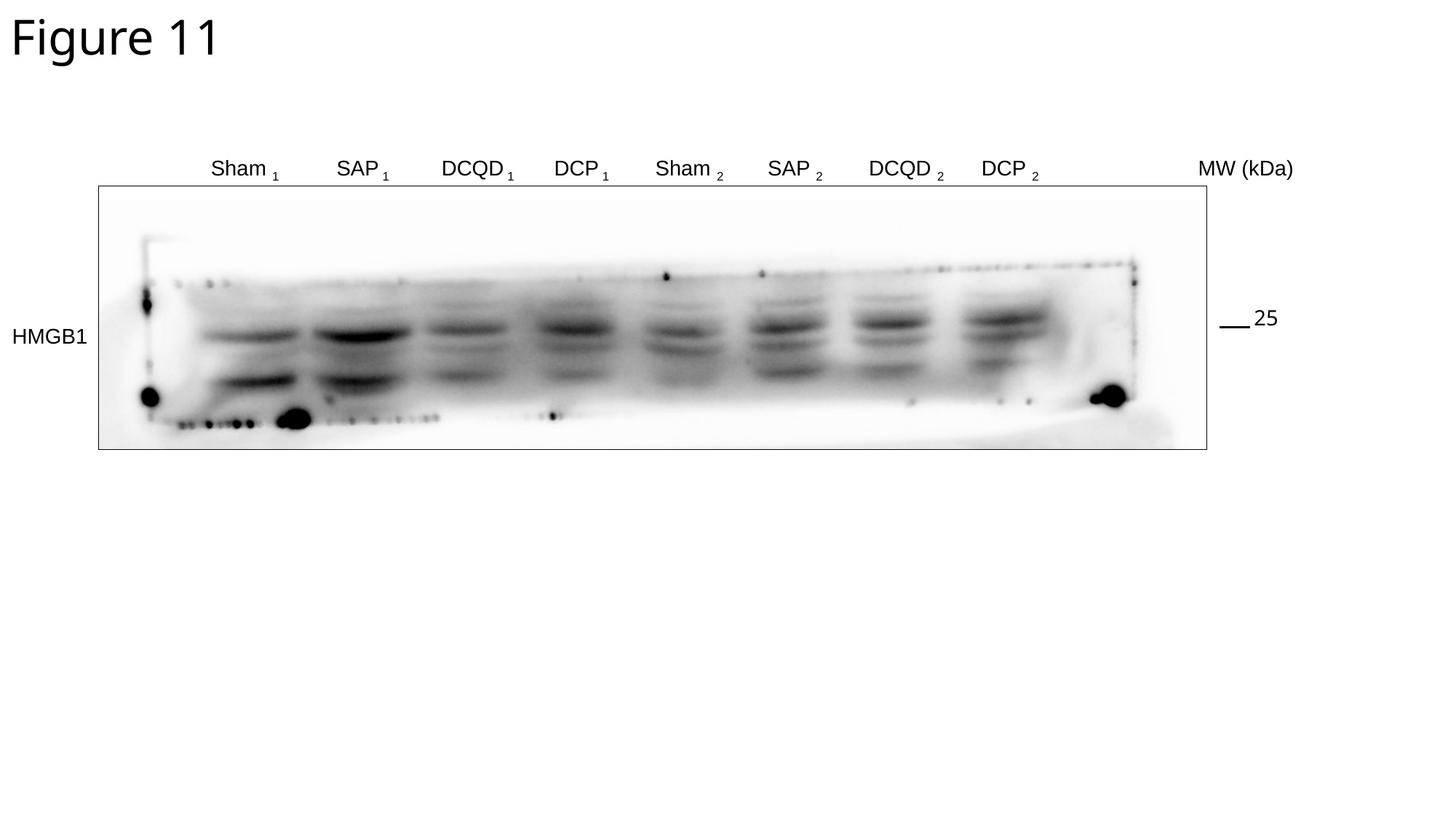

Figure 11
Sham 1
SAP 1
DCQD 1
DCP 1
Sham 2
SAP 2
DCQD 2
DCP 2
MW (kDa)
25
HMGB1

## Slide 16
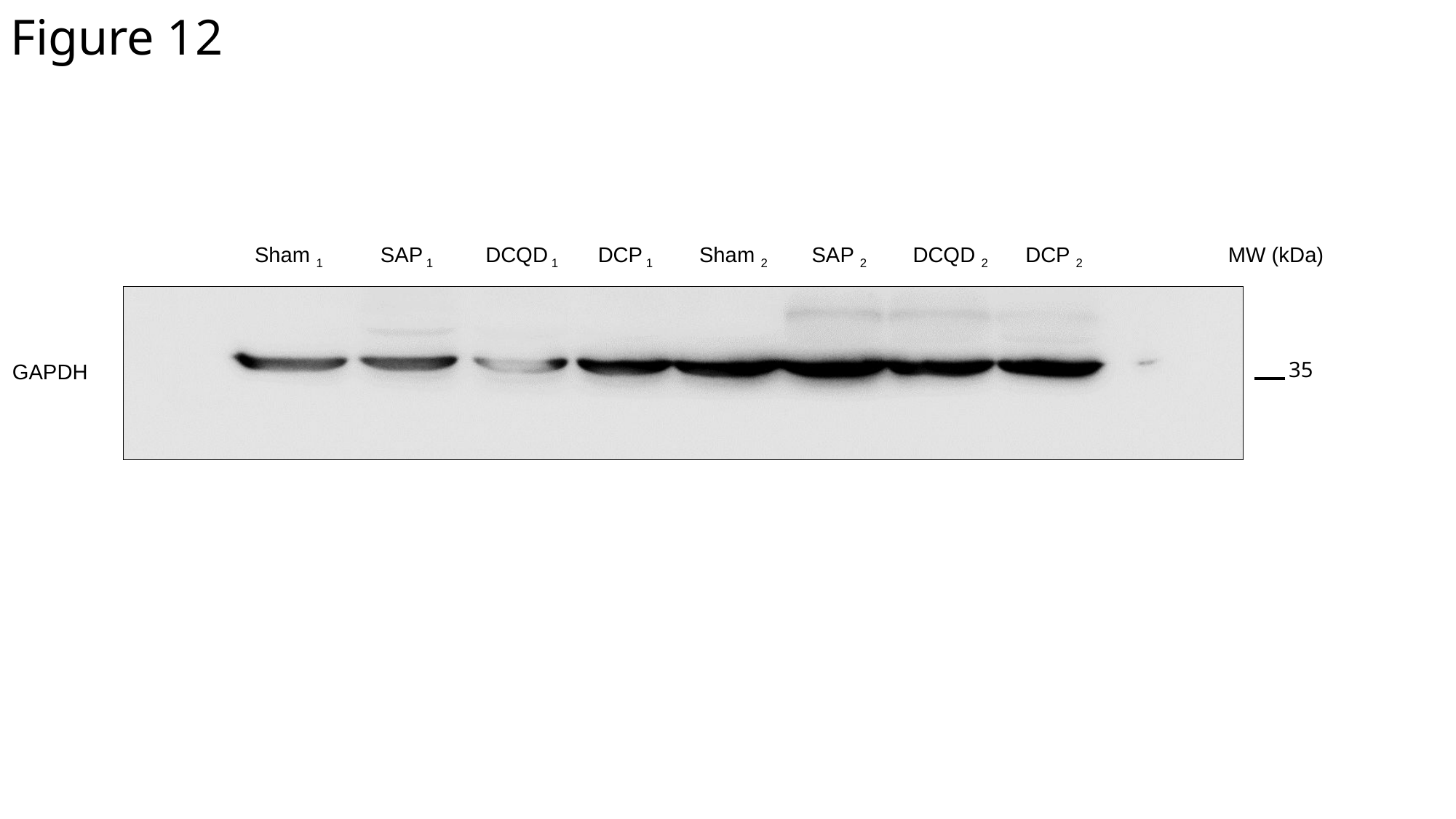

Figure 12
Sham 1
SAP 1
DCQD 1
DCP 1
Sham 2
SAP 2
DCQD 2
DCP 2
MW (kDa)
35
GAPDH
