# Supplementary material for: Dao-Chi Powder Ameliorates Pancreatitis-Induced Intestinal and Cardiac Injuries via Regulating the Nrf2-HO-1-HMGB1 Signaling Pathway in Rats
Source: Front Pharmacol. 2022 Jul 11;13:922130. doi: 10.3389/fphar.2022.922130 (PMC9310041; doi:10.3389/fphar.2022.922130)
Supplement: Supplementary file 1 [file DataSheet2.ZIP › additional files (Raw data)/Raw data sharing on Jianguoyun:Nutstore.docx]

Sorce data for H&E staining results: <https://www.jianguoyun.com/p/DR5xdUIQoLzAChjT07kEIAA>

Sorce data for molecular docking results:

<https://www.jianguoyun.com/p/DXWAbtoQoLzAChj407kEIAA>
