# Supplementary material for: Dao-Chi Powder Ameliorates Pancreatitis-Induced Intestinal and Cardiac Injuries via Regulating the Nrf2-HO-1-HMGB1 Signaling Pathway in Rats
Source: Front Pharmacol. 2022 Jul 11;13:922130. doi: 10.3389/fphar.2022.922130 (PMC9310041; doi:10.3389/fphar.2022.922130)
Supplement: Supplementary file 2 [file DataSheet1.docx]

Supplementary Material

# Supplementary Tables and Figures

## Supplementary Table

**Table S1. Major components of Dao-Chi powder detected by HPLC-MS/MS in serum (‾X ± S).**

| Monomers | Groups | duodenum (ng/mL) | Colon (ng/mL) | Heart (ng/mL) |
| --- | --- | --- | --- | --- |
| Ajugol | 2.4 g/kg.BW | N.D. | N.D. | N.D. |
|  | 4.8 g/kg.BW | N.D. | N.D. | N.D. |
|  | 9.6 g/kg.BW | 1.91±0.7426 | 2.203±0.6493 | 1.529±1.294 |
| Oleanolic acid | 2.4 g/kg.BW | N.D. | N.D. | N.D. |
|  | 4.8 g/kg.BW | N.D. | N.D. | N.D. |
|  | 9.6 g/kg.BW | 1.148±0.4829 | 0.825±0.2638 | N.D. |
| Stigmasterol | 2.4 g/kg.BW | N.D. | N.D. | N.D. |
|  | 4.8 g/kg.BW | N.D. | N.D. | N.D. |
|  | 9.6 g/kg.BW | 25.37±21.59 | N.D. | N.D. |
| Chlorogenic acid | 2.4 g/kg.BW | N.D. | N.D. | N.D. |
|  | 4.8 g/kg.BW | N.D. | N.D. | N.D. |
|  | 9.6 g/kg.BW | 38.03±18.58 | 8.545±3.682 | 16.8±6.602 |
| Glycyrrhizic acid | 2.4 g/kg.BW | N.D. | N.D. | N.D. |
|  | 4.8 g/kg.BW | N.D. | N.D. | N.D. |
|  | 9.6 g/kg.BW | 10.24±2.315 | 11.06±3.628 | 11.03±3.874 |
| Liquiritigenin | 2.4 g/kg.BW | N.D. | N.D. | N.D. |
|  | 4.8 g/kg.BW | 0.564±0.4701 | N.D. | N.D. |
|  | 9.6 g/kg.BW | 1.446±1.089 | 0.8417±0.442 | N.D. |

N.D.: Not detected.

# Supplementary method

**(Supplementary note to Result 5)**

**Da-Cheng-Qi decoction (DCQD)** group: the experimental severe acute pancreatitis of rats was induced by retrograde injection of 3.5% taurocholic acid sodium salt (1 mL/kg.BW) into the biliopancreatic duct at a rate of 6 mL/h. Next, DCQD (9.6 mg/kg.BW) were administered intragastrically to rats 12 h after modeling. Duodenum, colon, and heart samples were harvested at 36 h after the operation for the Western blot and Real-Time PCR analysis.

### DCQD preparation

DCQD was first recorded in “Shang-Han-Lun” and is one of the four classics of Traditional Chinese Medcine, in which the described ratio of Dahuang: Houpu: Zhishi: Mangxiao is 12:15:12:9, and the detailed composition is shown in Table S2. According to the Pharmacopoeia of China (2010), the recommended dosage of DCQD is 48 g per person (60 Kg.BW). Based on the pharmacological methodology, rats could be given 6 ~ 24 × of the human dosage (Zhang et al., 2017). As described previously, 12 × of the adult dose could effectively relieve the pathological damage of multiple organs in rats with severe acute pancreatitis (Li et al., 2015). Hence, we chose 12 × of the human dosage, meaning that the experimental dosage was 9.6 g/kg.BW. The spray-dried drug powders of DCQD used in the current experiment were sourced from Chengdu Green Herbal Pharmaceutical Co. Ltd. (Chengdu, China), which were extracted twice by refluxing with boiling distilled water (1:12, g/mL) for 1 h, and the obtained solution was concentrated and spray-dried. The dry powder was stored at 4 ℃ until use. Subsequently, the obtained spray-dried drug powders were mixed and reconstituted freshly with sterile double-distilled water (concentration: 0.96 g/mL) and administered orally to the rats at a dose of 1 mL/100 g.BW for once.

**Table S2. The ingredients list of Da-Cheng-Qi decoction**

| Ingredients (Pinyin, China) | Species | Weight (g) | Batch No. |
| --- | --- | --- | --- |
| Dahuang | Root and rhizome of *Rheum palmatum* L. | 12 | 18010003 |
| Houpo | Bark of *Magnolia officinalis* Rehder & E.H.Wilson | 15 | 17120120 |
| Zhishi | Fruit of *Citrus × aurantium* L. | 12 | 17100089 |
| Mangxiao | Mirabilite | 9 | 17060062 |

## Supplementary Figure


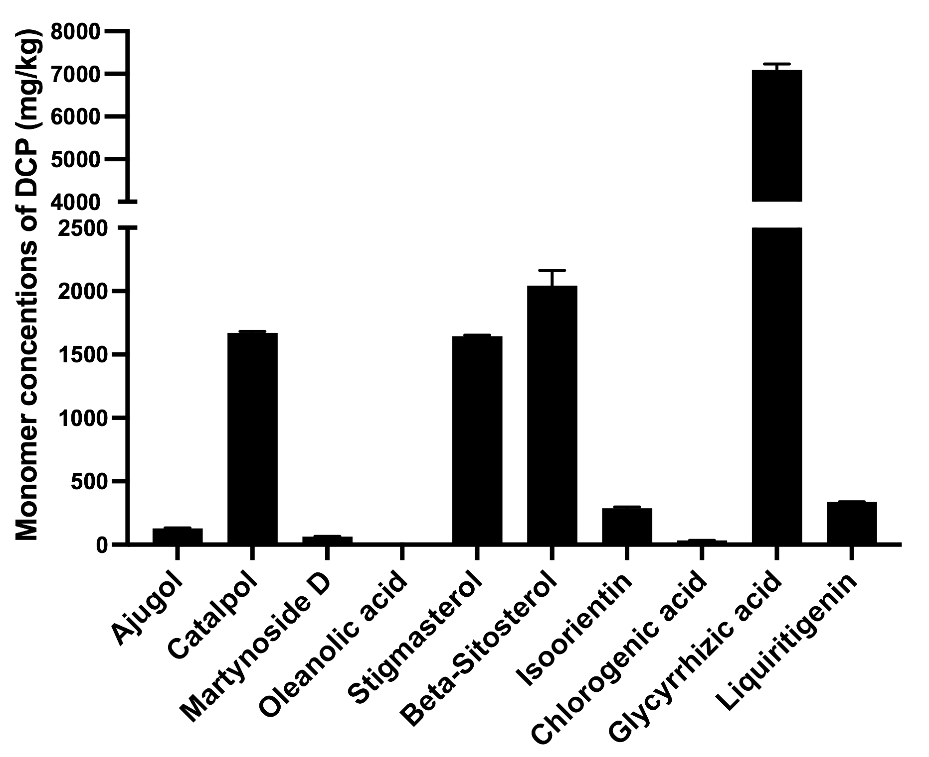


**Figure S1. Ten main monomers in Dao-Chi powder quantified by HPLC-MS/MS.**

# References

Li, J., Zhu, S.F., Zhao, X.L., Liu, Y.X., Wan, M.H., Guo, H., et al. (2015). Metabolomic profiles illuminate the efficacy of Chinese herbal Da-Cheng-Qi decoction on acute pancreatitis in rats. *Pancreatology* 15(4)**,** 337-343. doi: 10.1016/j.pan.2015.04.011.

Zhang, Y.M., Ren, H.Y., Zhao, X.L., Li, J., Li, J.Y., Wu, F.S., et al. (2017). Pharmacokinetics and pharmacodynamics of Da-Cheng-Qi decoction in the liver of rats with severe acute pancreatitis. *World J Gastroenterol* 23(8)**,** 1367-1374. doi: 10.3748/wjg.v23.i8.1367.
